# Implementation and First Evaluation of Strong-Correlation-Corrected Local Hybrid Functionals for the Calculation of NMR Shieldings and Shifts

**DOI:** 10.1021/acs.jpca.3c08507

**Published:** 2024-03-08

**Authors:** Caspar Jonas Schattenberg, Martin Kaupp

**Affiliations:** † Research Unit of Structural Chemistry & Computational Biophysics, Leibniz-Forschungsinstitut für Molekulare Pharmakologie (FMP), Robert-Roessle-Str. 10, 13125 Berlin, Germany; ‡ Institut für Chemie, Theoretische Chemie/Quantenchemie, Technische Universität Berlin, Sekr. C7, Straße des 17. Juni 135, D-10623 Berlin, Germany

## Abstract

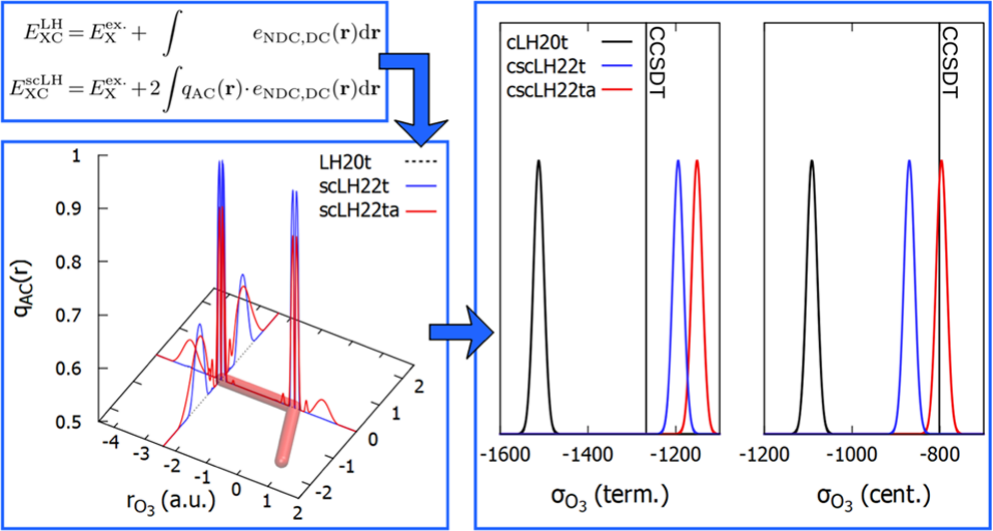

Local hybrid functionals containing strong-correlation factors (scLHs) and range-separated local hybrids (RSLHs) have been integrated into an efficient coupled-perturbed Kohn–Sham implementation for the calculation of nuclear shielding constants. Several scLHs and the ωLH22t RSLH have then been evaluated for the first time for the extended NS372 benchmark set of main-group shieldings and shifts and the TM70 benchmark of 3d transition-metal shifts. The effects of the strong-correlation corrections have been analyzed with respect to the spatial distribution of the sc-factors, which locally diminish exact-exchange admixture at certain regions in a molecule. The scLH22t, scLH23t-mBR, and scLH23t-mBR-P functionals, which contain a “damped” strong-correlation factor to retain the excellent performance of the underlying LH20t functional for weakly correlated situations, tend to make smaller corrections to shieldings and shifts than the “undamped” scLH22ta functional. While the latter functional can also deteriorate agreement with the reference data in certain weakly correlated cases, it provides overall better performance, in particular for systems where static correlation is appreciable. This pertains only to a minority of systems in the NS372 main-group test set but to many more systems in the TM70 transition-metal test set, in particular for high-oxidation-state complexes, e.g., Cr(+VI) complexes and other systems with stretched bonds. Another undamped scLH, the simpler LDA-based scLH21ct-SVWN-m, also tends to provide significant improvements in many cases. The differences between the functionals and species can be rationalized on the basis of one-dimensional plots of the strong-correlation factors, augmented by isosurface plots of the fractional orbital density (FOD). Position-dependent exact-exchange admixture is thus shown to provide substantial flexibility in treating response properties like NMR shifts for both weakly and strongly correlated systems.

## 1 Introduction

In view of the central importance of nuclear magnetic resonance (NMR) spectroscopy in many areas of natural science, the quantum chemical calculation of NMR parameters is an active and important field in its own right.^1^ As for many other areas of electronic structure calculations, Kohn–Sham density functional theory (KS-DFT) provides the main work horse also in NMR-parameter computations due to its favorable cost-performance ratio. In the field of nuclear shielding computations and the derived NMR chemical shifts, on which this work concentrates, the dependence on the approximate exchange–correlation (XC) functional used can be very large, however. Hierarchies like Perdew’s “Jacob’s ladder”^2^ alone do not allow one to predict which density functional approximation (DFA) will perform best for shielding calculations. For this reason, systematic benchmark studies have to be used to select suitable functionals for chemical applications. Many nuclear-shielding/chemical-shift benchmarks have been published over the years (see, e.g., refs (3−15) for main-group nuclei and refs (16−25) for transition-metal nuclei). Some of them rely on experimental data and some on computed high-level post-Hartree–Fock ab initio data for small molecules (often at CCSD(T) coupled-cluster level).

We have recently reported two particularly large and representative benchmark sets: the NS372 set for shieldings/shifts of light main-group molecules based on CCSD(T)/pcSseg-3 reference data^26^ and the TM70 set of 3d transition-metal nucleus shifts,^27^ based on the experimental data. These two sets have been applied to the evaluation of a wide variety of DFAs, including recent implementations of local hybrid functionals (LHs) with position-dependent exact-exchange (EXX) admixture,^28−30^ as well as meta-GGA functionals with inclusion of the proper current-density response for local kinetic energy,^29^ that had not been dealt with correctly in previous implementations.

Some of the insights obtained from the extensive benchmark studies for the NS372 set are as follows.^26^ (a) The top performance of the DSD-PBEP86 double-hybrid functional (DH) and the overall rather good performance of MP2 calculations found in previous smaller benchmark evaluations were confirmed. (b) For this main-group set, several recently implemented LHs also performed very well, in particular with the proper inclusion^29,30^ of the current-density response. (c) With the current-density response accounted for properly, several highly parametrized meta-GGA functionals were also among the top performers, in particular, cB97M-V and cMN15-L (prefix “c” indicates the inclusion of current-density response). (d) Larger deviations are found for a number of molecules in the set, for which static correlation effects are more important. However, given the construction of the set based on CCSD(T) reference data, only a few of these systems are included in the statistical evaluation of DFAs for NS372 or other main-group shielding benchmarks.

Matters are different for the TM70 3d-shift benchmark:^27^ (a) here, double hybrids or MP2 are not applicable due to static correlation playing a major role for most subsets (only ^49^Ti shifts were found to be slightly less sensitive). Indeed, single-reference coupled-cluster theory is not expected to be accurate, and the size of the systems precludes the setup of a purely theoretical benchmark in this area. (b) Several LHs were among the best-performing DFAs, albeit with a slight preference for overall less EXX admixture compared to the main-group case. (c) Upon inclusion of the current-density response, several parametrized meta-GGAs again performed well, particularly cM06-L and cB97M-V. These studies also confirmed earlier findings that, while moderate amounts of EXX admixture are favorable for, e.g., ^57^Fe or ^59^Co shifts,^16−20^ constant, position-dependent or range-separated EXX admixture actually enhances deviations from the experiment in other cases, in particular^21^ for ^53^Cr shifts.^27^ It seems clear that both static correlation errors (fractional spin errors, FSE^31−33^) and self-interaction errors (delocalization errors, fractional-charge errors, FCEs^31,33,34^) have to be minimized to improve the performance further for such challenging transition-metal shifts. While several functionals achieve percentage mean absolute errors below 1.5% for the NS372 main-group shielding and shift benchmark, for the TM70 set, the best functionals so far just achieve about 4–5%. There is thus still significant room for improvement in shielding/shift computations, in particular for transition-metal complexes (here, we focus on the metal nuclei, but the metal-bonded ligand-nucleus shifts are also expected to be affected strongly by similar shortcomings).

In this context, it is important to note the often bemoaned zero-sum game between minimizing FSEs and FCEs.^35,36^ That is, increasing EXX admixture helps to reduce FCEs but typically increases FSEs, and vice versa. An escape from this zero-sum game, i.e., finding functionals with low self-interaction/delocalization as well as static correlation errors is arguably the most challenging goal of contemporary developments of DFAs. The recent contributions of our own group to this endeavor have focused on the augmentation of LHs by so-called strong-correlation (sc) factors, providing scLHs. Taking clues from other important coordinate-space models of nondynamical and strong correlation, such as the B13^37^ and KP16/B13^38^ functionals, we have constructed a number of scLH models,^39−41^ in particular on the basis of the LH20t functional.^42^ The scLH22t and scLH22ta functionals^40^ and the earlier, simpler LDA-based model scLH21ct-SVWN-m^39^ adapted much of the KP16/B13 machinery in constructing the sc-factor *q*_AC_(**r**). The more recent^41^ scLH23t-mBR and scLH23t-mBR-P functionals use a simplified approach, where the real-space functions determining local static correlations are based on a simple ratio of semilocal exchange and EXX energy densities. scLH22t, scLH23t-mBR, and scLH23t-mBR-P contain damping factors within *q*_AC_(**r**) that avoid double counting of static correlations for more weakly correlated situations and largely retain the excellent performance (and the underlying parameters) of LH20t in such cases. scLH22ta and scLH21ct-SVWN-m lack such damping and may bring in more of the strong-correlation contributions while somewhat deteriorating performance in more weakly correlated situations.^39,40^

Here, we report the first implementation of scLHs for nuclear shieldings, thereby extending the existing implementation^28−30^ for LHs in the Turbomole program suite.^43−45^ We evaluate scLHs for the above-mentioned diverse and extended NS372 and TM70 shielding/shift benchmark sets. Additionally, we have also implemented and will evaluate for the first time for nuclear shieldings the recent successful range-separated LH (RSLH) ωLH22t, which has been shown to perform excellently for a wide variety of questions and which exhibits the correct long-range asymptotic potential.^46,47^ We note in passing that recent scRSLH extensions of ωLH22t^48^ are not yet covered here and will be reported elsewhere. A related, first successful implementation and evaluation of scLHs and RSLHs for a benchmark set of magnetizabilities has just been reported.^49^ The implementation shares parts of the code with the shielding code; here, we will give more of the theoretical background.

The presentation is organized as follows: we introduce the basic concepts of local hybrid functionals and their extension to range-separation and strong correlation in Section 2, followed by a description of the relevant equations for NMR theory and for the implementation of the novel functionals in a coupled-perturbed scheme (Section 3) and a description of the relevant computational details (Section 4). Section 5 gives a detailed analysis of the performance of the new functional models for the NS372 and TM70 benchmark sets, followed by conclusions (Section 6).

## 2 Theory

### 2.1 Local Hybrid Functionals, LH20t

LHs replace the constant EXX admixture of global hybrids (GHs)^50,51^ by a position-dependent local mixing function (LMF, *g*(**r**)).^52^ The energy expression of such an LH can be written compactly as
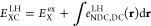
1where *E*_X_^ex^ is the (integrated) EXX energy and *e*_NDC,DC_^LH^(**r**) describes a nondynamical (NDC) and dynamical correlation (DC) contribution, which for a general LH can be written as
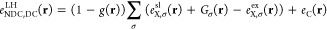
2where *e*_C_ is the semilocal (dynamical) correlation energy density, *e*_X,σ_^sl^(**r**) is the semilocal exchange, and *e*_X,σ_^ex^(**r**) is the EXX energy density, given in the basis of the atomic orbitals (AOs) ϕ by
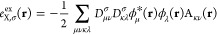
3where *D*^σ^ are density matrices for spin σ in the AO basis and
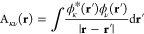
4is the so-called A-matrix occurring in the seminumerical integration of the LH EXX terms.^53−58^ Various models have been proposed for the LMF *g*(**r**) (see ref (52). for a review). We will in the following concentrate on the most widely used t-LMF type constructed as the scaled ratio of the von-Weizsäcker and Kohn–Sham kinetic-energy densities^59−62^
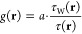
5with
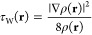
6

*G*_σ_(**r**) is a calibration function (CF) introduced to correct for the ambiguity of the exchange-energy densities.^63−65^ We will focus on CFs constructed via integration by parts from a semilocal exchange functional within the second-order partial-integration-gauge (pig2) scheme,^65^ with
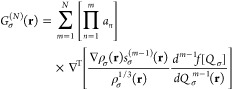
7*N* is the order of the correction, i.e., *N* = 2 for pig2, {*a*_*n*_} constitutes a set of (semi)empirical parameters, ρ(**r**) is the electron density, and *s*_σ_(**r**) is the dimensionless reduced gradient of the density
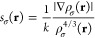
8*Q*_σ_(**r**) is the inhomogeneity parameter used in the underlying exchange enhancement factor, *f*[*Q*_σ_], of the semilocal functional. A pig2-CF has the general form
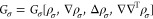
9constructed solely from semilocal quantities. For convenience, we denote
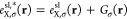
10Most of the scLHs discussed in this work are based on LH20t,^42^ which employs a *t*-LMF, PBE exchange,^66^ a pig2-CF,^65^ and a B95^67^ DC part.

### 2.2 Range-Separated Local Hybrid Functionals, ωLH22t

Starting from eq 1, an RSLH functional^68−70^ can be expressed by a reformulation of the NDC/DC contribution using the range-separated EXX energy density^46^
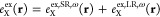
11where the short- and long-range (SR, LR) contributions are separated by substitution of the Coulomb operator, using
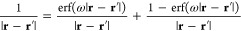
12where ω is the range-separation parameter. The (nondynamical and dynamical) correlation contribution *e*_NDC,DC_^ω^(**r**) is then given by
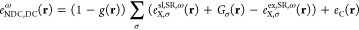
13with *e*_X_^sl, SR,ω^(**r**) being the short-range semilocal exchange-energy density. The ωLH22t RSLH^46^ covered in this work is a relatively straightforward extension of LH20t based on short-range PBE exchange, a t-LMF, a pig2-CF, and a B95 DC correlation. Exemplarily, the short-range EXX energy density can be written as
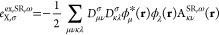
14with the short-range contribution of the full-range A-matrix (A_κν_(**r**) = A_κν_^SR,ω^(**r**) + A_κν_^LR,ω^(**r**)) given by
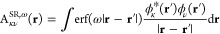
15

### 2.3 Strong-Correlation-Corrected Local Hybrid Functionals

scLHs apply an sc-factor *q*_AC_(**r**) to the NDC and DC terms of an LH to recover the kinetic-energy correlation contribution from a local version of the adiabatic connection (AC), thereby improving the description of strong correlations, e.g., when computing spin-restricted bond dissociation curves or generally minimizing FSEs. Compared to eq 1, this brings the substitution *e*_NDC,DC_(**r**) → *e*_NDC,DC_^sc^(**r**), where
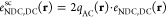
16analogous to the introduction of a related factor *q*_AC_(**r**) (only) to the NDC term in the KP16/B13 coordinate-space model.^38^ Note that the underlying LH is restored by setting *q*_AC_(**r**) = 0.5 everywhere in space.

Different forms of *q*_AC_(**r**) have meanwhile been evaluated. The first LDA-based, simple scLH, scLH21ct-SVWN-m,^39^ and the first of the more sophisticated LH20t-based models, scLH22a, remained close to the KP16/B13 idea, where the local AC interpolation is formulated as^38^
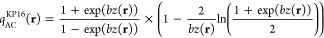
17where *b* is a semiempirical parameter, and the local function
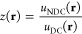
18is constructed from the nondynamical (*u*_NDC_) and dynamical (*u*_DC_) correlation potentials. *z*(**r**) determines the importance of NDC in real space and is computed from the exchange-hole normalizations of the reverse Becke–Roussel machinery underlying both B13 and KP16/B13 models (with the small modification^39,40^ of using a modified expression, mB13, based on Patra’s hole curvature^71^).

In ref (40), an additional model, scLH22t, was proposed, which applies an exponential damping factor to function *z*(**r**) to further reduce small values and thus avoid double counting of NDC contributions in weakly correlated situations,
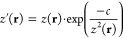
19(*c* is an adjustable parameter). This allowed scLH22t to retain the underlying parameters of LH20t without modification and gave an almost unchanged performance compared to the LH for test sets of weakly correlated systems like the extended GMTKN55 main-group energetics suite.^40^

In the most recent scLH models,^41^ the complicated reverse Becke–Roussel machinery of B13 or KP16/B13 has been replaced by a simpler comparison of semilocal and exact-exchange-energy densities (eq 20) and by modified local AC interpolation formulas. Here, we will evaluate scLH23t-mBR and the somewhat more involved scLH23t-mBR-P model. Both construct *z*(**r**) by comparing a modified (but not reverse) Becke–Roussel exchange-energy density^72^ (also with Patra’s hole curvature,^71^ see above) with the EXX one
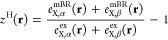
20

scLH23t-mBR uses an error-function local AC interpolation
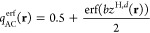
21(*b* is an adjustable parameter), and an error-function-damped form of *z*(**r**)
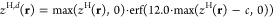
22(*c* is also an adjustable parameter).

scLH23t-mBR-P has been constructed^41^ to decrease the small unphysical local maxima found for other models in the spin-restricted potential-energy curves at intermediate distances in some diatomic molecules and is based on a more complicated Padé form
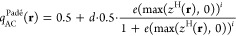
23Parameters *d*, *e*, and *i* are fitted to MRCI + Q and LH data for the N_2_ dissociation curve: damping for weakly correlated situations is achieved by reproducing the uncorrected LH20t-based curve around the minimum while taking into account the MRCI + Q energies for longer distances. This form has built-in damping and thus employs the undamped *z*^H^(**r**).

For the implementation described in the next section, it is important to note that the sc-factor has the general form
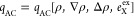
24That is, in contrast to the semilocal t-LMF and CF, it depends explicitly on the EXX energy density.

## 3 Implementation of scLHs and RSLHs for NMR Shielding Constants

### 3.1 Calculation of Shielding Tensors for Global and Local Hybrid Functionals

The tensor components of the NMR shielding constant for nucleus *K* with the Cartesian indices *k* and *l* can be obtained by perturbation theory as the second derivative with respect to the external magnetic field **B** and nuclear magnetic moment **M**_*K*_,
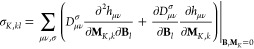
25To solve eq 25, the perturbed density has to be constructed in atomic orbital (AO) representation as
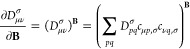
26where *D*_*pq*_^σ^ is the molecular-orbital (MO) density matrix defined as *D*_*ij*_^σ^ = δ_*ij*_; *D*_*ia*_^σ^ = *D*_*ai*_^σ^ = *D*_*ab*_^σ^ = 0 (indices *i*,*j*,··· denote occupied, *a*,*b*,··· virtual and *p*,*q*,··· general MOs). eq 26 can be calculated from the perturbed MO coefficients, expanded in the set of MO coefficients of the virtual states weighted by the expansion coefficients *u*_*pq*_ as
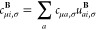
27Finally, *u*_*pq*_ can be obtained from the coupled-perturbed equations
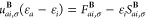
28which depend on the chosen DFA via the perturbed Fock contribution, *F*_*ai*,σ_^**B**^ = *F*_*ai*,σ_^**B**^[···, (*V*_*ai*,σ_^XC^)^**B**^], where
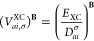
29is the perturbed XC potential.

Throughout this work, gauge-including atomic orbitals (GIAOs)^73−75^ are employed, written as
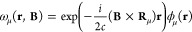
30where **R**_μ_ is the position of the basis function, i.e., typically the position of the nucleus on which ϕ_μ_(**r**) is centered. The use of these basis functions ensures fast convergence of the shielding results to the basis-set limit to obtain results that are unaffected for all practical purposes by the gauge origin (**R**_G_) of the magnetic vector potential
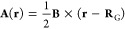
31used to describe the external magnetic field.

As shown in eq 29, the crucial step for the implementation of DFAs for the calculation of shielding constants is the derivation of the perturbed potential (full expressions for the perturbed potentials of LHs RSLHs and scLHs are given in Section S1 of the Supporting Information). For LH functionals, a detailed description of the implementation can be found in ref (28). Extensions of the implementation cover higher derivatives of the density,^30^ involved when including semilocal CFs, and the Dobson current-density response for the kinetic-energy density.^29^ The latter has the benefit of inducing a proper current-response of the semilocal exchange–correlation contributions of τ-dependent functionals (e.g., meta-GGAs and many LHs) and thus circumventing artifacts plaguing the widespread model by Maximoff and Scuseria.^76^ Here, we will recap only briefly the central equations for LHs that are important for understanding the following exposition. The perturbed LH potential can be separated into contributions dependent on EXX and semilocal contributions. The EXX-dependent contribution is then given by
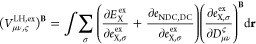
32where for LHs simply
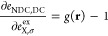
33and
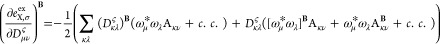
34A crucial step is the construction of the perturbed A-matrix, given by
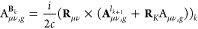
35Here, **A**_μν,*g*_^*l*_*k*+1_^ is a vector composed of the three A-matrices with an enlarged azimuthal quantum number (*l*_*k*_) of the respective Gaussian basis function with Cartesian component *k* and the index *g* describes a component of the numerical integration grid (see below). In contrast to GH functionals, the construction of **A**_μν,g_^*l*_*k*+1_^ cannot be (conveniently) circumvented for LHs due to the presence of the LMF (see eq 32). Also, due to the LMF, LHs are also most conveniently implemented in a seminumerical fashion (for details, see ref (28) for the shielding case or ref (52) for a general discussion in the context of LHs), i.e., the “outer” integration in the 4-center integrals is replaced by a quadrature on a numerical grid, and just the “inner” A-matrices are solved analytically (hence the dependence on the grid components). This allows a stepwise contraction, first of the basis-function vectors with the density matrices, giving the F-vectors
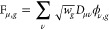
36
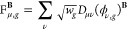
37and
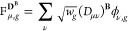
38where  is a grid weighting factor. Second, the resulting F-vectors are contracted on the fly with the A-matrices, avoiding the need to store the latter explicitly. The three G-vectors to be constructed are then
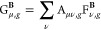
39
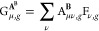
40and
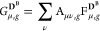
41Clearly, eq 41 depends on the perturbed density and is thus solved within the CPKS-loops, whereas the other G-vectors of eqs 39, 40 have to be constructed just once in the pre-CPKS part of the program. The semilocal part reads
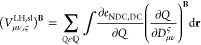
42where  contains the semilocal quantities present in the semilocal exchange and correlation energy densities, as well as the CF and LMF used in the chosen functional. Exemplarily,
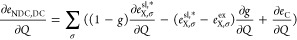
43then describes the derivative with respect to the semilocal quantity *Q*.

### 3.2 Extension of the Shielding Code to Range-Separated Local Hybrids

Based on the implementation of LH functionals, the extension of the shielding implementation to RSLH functionals requires the separation of the exact-exchange contribution into its SR and LR parts (eq 11). Conveniently, the derivatives can be separated, giving
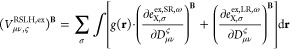
44where the respective density matrix derivatives of the SR and LR EXX contributions are constructed by substitution of the A-matrix with its SR and LR equivalents, as shown in eq 34. Hence, the perturbed SR and LR A-matrices have to be constructed. Similarly to LH functionals (eq 35), these can be calculated using A-matrices with an enlarged *l*-quantum number via
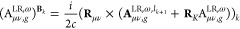
45and
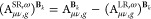
46The extension thus requires the calculation of two separate perturbed A-matrices. In this implementation, we construct these terms from the full-range and LR A-matrices, as the associated integrals were already available in the program code.^46^ The final contraction of the exact-exchange contributions then requires the construction of the three G-vectors,
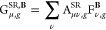
47
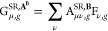
48
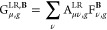
49to be constructed in the preloop part, and two G-vectors in the CPKS-loops
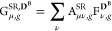
50
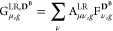
51Note that for the LR part, two additional G-vectors suffice, as the LR contribution is not scaled by the LMF.

### 3.3 Extension of the Shielding Code to Strong-Correlation-Corrected Local Hybrids

The implementation of scLHs is based on eq 32 for the EXX contributions and eq 42 for the semilocal contributions. The substitution of *e*_NDC,DC_^LH^ by *e*_NDC,*DC*_^sc^ then leads to the derivatives
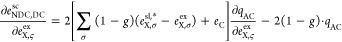
52and
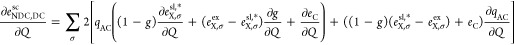
53which are finally contracted with the perturbed semilocal and EXX-dependent integrals as implemented for LHs. We note that the inclusion of the sc-terms into LHs adds negligible additional computational demands to either ground-state SCF or shielding calculations (data not shown; we refer to ref (28) for a thorough investigation on the timings of LHs in comparison to other functionals in shielding calculations).

## 4 Computational Details

### 4.1 Computational Setup

Unless stated otherwise, calculations with the newly implemented scLH and RSLH functionals have been performed using a local developers’ version of the Turbomole program code based on version 7.7.^43−45^ Results with other functionals have been taken for comparison from refs (26,27). The new implementation has been carefully tested by ensuring gauge independence and comparison of limiting cases for RSLHs (lim_ω→∞_*E*_XC_^RSLH^ = *E*_X_^ex^ + *E*_C_; lim_ω→0_*E*_XC_^RSLH^ = *E*_XC_^LH^), as well as for scLHs (lim_*q*_AC_→0.5_*E*_XC_^scLH^ = *E*_XC_^LH^). For all calculations, energy convergence was set to 10^–9^ while additionally ensuring convergence of the density matrix using the internal keyword denconv with the setting 10^–7^. Convergence of the Euclidian norm of the residual vectors in the CPKS shielding evaluation was set to 10^–7^. The resolution-of-the-identity approximation (RIJ) was used for the Coulomb operator within the SCF and shielding calculations for NS372 data.^90^ Unless noted otherwise, grids of size “3”^91^ (internal Turbomole setting) and pcSseg-3 basis sets,^92^ which are known to be close to the basis-set limit for DFT shielding computations,^26^ have been used. Gauge independence of the kinetic-energy density in the CPKS equations was ensured^26,27,29,30^ using the Dobson formulation^93,94^ of τ, which renders the functionals implicitly current-dependent. In line with previous studies,^26,27,80,95−97^ the prefix “c” is added to the name of the DFAs when pointing to the shielding results with the current-density response. Previous evaluations for the NS372 benchmark with double-hybrid functionals^26^ were done with the Orca program package, version 4.2.1,^12,13,98,99^ using settings as suggested in ref (13). GIAO coupled-cluster results with perturbative and full triples (CCSD(T) and CCSDT) were calculated using the cfour program package, version 1.2.^100^

Besides the newly implemented scLH and RSLH models, we include some of the best-performing functionals from the former studies, as listed in Table 1. We note in passing that the response of the VV10 dispersion correction^101^ (as used in cB97M-V, ωB97X-V, and cωB97M-V) is not included in the CPKS equations of Turbomole. Hence, the dispersion part of the functional only directly affects the ground-state SCF, while the shielding is influenced only indirectly via the altered ground-state density.

**Table 1:** Exchange–Correlation Functionals Evaluated in This Work

functional	type	notes	references
DSD-PBEP86	DH		(77,78)
scLH23t-mBR-P^a^	scLH	ct-LMF (*a* = 0.715), damped *q*_AC_^Padé^, pig2-CF, X_0.22S+0.78PBE_ + C_modB95_	(41)
scLH23t-mBR^a^	scLH	ct-LMF (*a* = 0.715), damped *q*_AC_^erf^, pig2-CF, X_0.22 S+0.78 PBE_ + C_modB95_	(41)
scLH22t	scLH	ct-LMF (*a* = 0.715), damped *q*_AC_^KP16^, pig2-CF, X_0.22 S+0.78 PBE_ + C_modB95_	(40)
scLH22ta	scLH	ct-LMF (*a* = 0.766), *q*_AC_^KP16^, pig2-CF, X_0.04 S+0.96 PBE_ + C_modB95_	(40)
scLH21ct-SVWN-m	scLH	ct-LMF (*a* = 0.628), *q*_AC_^KP16^, X_S_ + C_VWN_	(39)
ωLH22t	RSLH	ct-LMF (*a* = 0.587), ω = 0.233, pig2-CF, X_SR–PBE_ + C_modB95_	(46)
LH20t	LH	ct-LMF (*a* = 0.715), pig2-CF, X_0.22 S+0.78 PBE_ + C_modB95_	(42)
LH14t-calPBE	LH	t-LMF (*a* = 0.5), pig1-CF, X_0.49 S+0.51 PBE_ + C_0.55 PW92+0.45 PBE_	(64)
LH12ct-SsirPW92	LH	ct-LMF (*a* = 0.646), X_S_ + C_sirPW92_	(62)
mPSTS-noa2	LH	modified PSTS LH, without *a*_2_ part of LMF^b^	(79,80)
B3LYP^c^	GH		(51,81,82)
ωB97M-V	RSH, mGGA		(83)
ωB97X-V	RSH, GGA		(84)
B97M-V^d^	mGGA		(85)
VSXC^d^	mGGA		(86)
M06-L	mGGA		(87)
KT2^d^	GGA		(88)

a Simplified *q*_AC_ constructions based on ratios between mBR and EXX energy densities with error function (scLH23t-mBR) or Padé form (scLH23t-mBR-P); see ref (41) for details.

b See ref (80) for further details of the noa2 model.

c Used in its VWN5 variant, as implemented in Turbomole.

d Used via the Libxc library.^89^

Fractional occupation number densities (FODs) have been calculated using Grimme’s FODplot tool for Turbomole^102^ at the TPSSh/pcSseg-3 level for main-group compounds and at the TPSSh/pcSseg-2 level for transition-metal complexes using the default electronic temperature of 5000 K. The resulting isosurfaces, as well as the definition vectors for the *q*_AC_(**r**) plots, were computed using VMD version 1.9.1.^103^

### 4.2 scLH and RSLH Functionals

The first evaluation of scLHs in shielding calculations in this work includes five functionals from refs (39−41). While four of the functionals (scLH22t,^40^ scLH22ta,^40^ scLH23t-mBR,^41^ and scLH23t-mBR-P^41^) are based on the more advanced LH20t functional,^42^ scLH22ct-SVWN-m is a simpler construction^39^ based on LDA exchange and without calibration of exchange-energy densities. An important distinction between these scLHs in the present context is also, however, that scLH22t, scLH23t-mBR, and scLH23t-mBR-P contain damping factors within their *q*_AC_ functions to ensure essentially unchanged LH20t parameters and unaltered performance for weakly correlated situations (see Section 2), while scLH22ta and scLH22ct-SVWN-m do not. We will, in the following, label the former three scLHs as “damped” and the latter two as “undamped”. ωLH22t^46^ is included as the only RSLH.

### 4.3 Test Sets for NMR Shieldings and Shifts

NMR shifts are calculated as
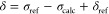
54where σ_calc_ and σ_ref_ are the NMR shielding constants of the target species and reference compound, as obtained from eq 25. δ_ref_ is the corresponding shift of a secondary standard if used.

The NS372 benchmark set^26^ contains subsets with CCSD(T)/pcSseg-3 shielding/shift data for ^1^H, ^11^B, ^13^C, ^15^N, ^17^O, ^19^F, and a combined ^31^P and ^33^S subset, with 124, 14, 93, 43, 31, 47, and 20 entries, respectively. Shifts are referenced against CH_4_ (^1^H, ^13^C), BF_3_ (^11^B), NH_3_ (^15^N), H_2_O (^17^O), HF (^19^F), PH_3_ (^31^P), and H_2_S (^33^S).

The TM70 set^27^ is composed of shift subsets for ^49^Ti, ^51^V, ^53^Cr, ^55^Mn, ^57^Fe, ^59^Co, and ^61^Ni, with 12, 10, 10, 11, 9, 9, and 9 data points, respectively. Here, we use the Y-intercepts of the linear regressions of computed σ_iso_ against experimental δ_iso_ values at the respective computational levels as references to compute shifts from the shielding data. We note in passing that a careful examination in ref (27) included an estimate of environmental and (scalar-)relativistic effects, which were found to be small or negligible in the overall statistical comparison of experimental and calculated data.

As in our previous work,^26,27^ we present, in particular, statistical quantities in the main text, i.e., standard deviation (SD), mean absolute (MAE), and mean signed errors (MSE), as well as maximal relative absolute errors (max. rel. AE). We will denote the latter also as maximum percentage unsigned deviations. For the TM70 set, also the slopes of the linear regression lines are included. To obtain cross-nucleus comparability, the statistical measures are normalized to the ranges covered by the reference data for the respective nuclei, providing relative (percentage) deviations. Aggregate weighted percentage deviations are marked with “all” for both NS372 (with and without including the ^1^H shielding/shift data) and TM70. Detailed shielding and shift data are available in the Supporting Information.

## 5 Results

For both NS372 main-group shieldings and shifts^26^ and TM70 transition-metal shifts,^27^ performance of the new scLH and RSLH functionals is compared to a number of the best previously evaluated functionals. In particular, we include the LH14t-calPBE, LH12ct-SsirPW92, and LH20t functionals (most scLHs are based on LH20t, while scLH21ct-SVWN-m is most closely comparable to LH12ct-SsirPW92), and we also include the mPSTS LH without its *a*_2_-term (“noa2”) due to its pronounced potential for error compensation in relative main-group shifts^26^ (see below). Additionally, we include the best-performing mGGAs for the two benchmarks (B97M-V, VSXC, and M06-L). Note that the highly parametrized MN15-L mGGA also performed well for this test set^26^ but was completely unsuitable for the TM70 set, and we therefore do not discuss it here. However, we decided to include the overall best-performing functional for main-group shieldings, the double-hybrid DSD-PBEP86, even though it is also not suitable for the transition-metal series. KT2 is additionally included as an example of a DFA specifically designed for the calculation of main-group NMR shielding and shift data. The ωB97X-V and ωB97M-V RSHs are also included to compare to the ωLH22t RSLH. Table 1 gives an overview of the functionals. In the reported shielding data below, we will denote the inclusion of current-density response for τ-dependent functionals by the commonly used^26,27,80,95−97^ prefix “c”.

### 5.1 NS372 Shielding Test Set, Statistical Evaluation

**Figure 1:**
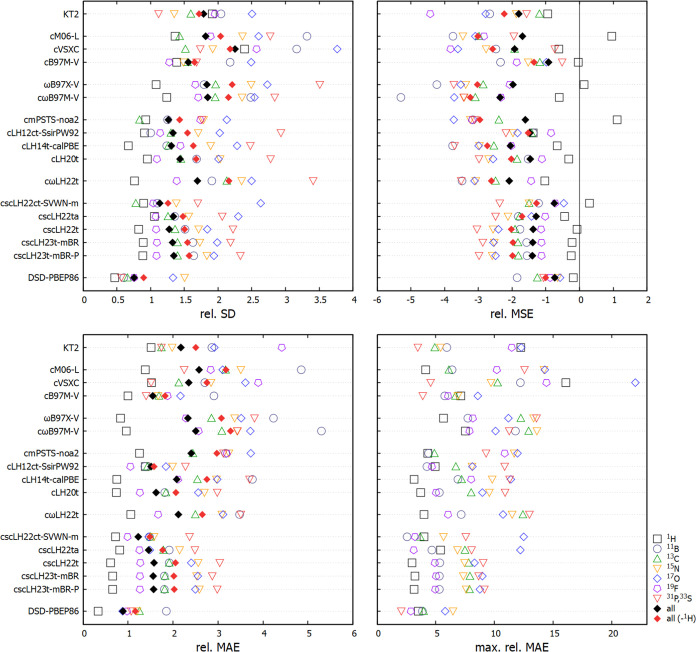
Statistical comparison of different functionals for the NS372 shielding test set. Percentage relative deviations are shown.

We first look at the main-group shieldings, where we expect the effects of strong correlation to be important only for a few cases. We nevertheless start with an overall statistical bird’s eye view, as provided in Figure 1. The ranges of the plots are determined by the largest deviations for the various nuclei for the functionals included in the survey, all taken relative to the GIAO–CCSD(T)/pcSseg-3 reference data. As done previously,^26^ several larger outliers with large statical correlation effects and/or extremely large paramagnetic contributions are excluded from the statistical evaluations, such as the ^17^O shieldings of O_3_, ^19^F shieldings of F_3_^–^, or the ^11^B shielding of BH. As such cases are particularly interesting in the context of scLH performance, we will discuss them separately further below, together with selected examples from within the NS372 set.

As found before,^26^ in the overall aggregate deviations, the SCS-MP2-based double-hybrid DSD-PBEP86 is the top-performing functional (rel. MAE slightly below 0.9%). We note in passing the much larger computational effort involved in the shielding computations with DHs or MP2 compared to functionals up to rung 4. Interestingly, we find the two undamped scLHs to give the next lowest rel. MAEs (csLH22ct-SVWN-m 1.2%, cscLH22ta <1.5%). This may be compared to the best performers up to rung 4 so far, namely, the cLH12ct-SsifPW92 LH and the meta-GGA cB97M-V (both near 1.5%). Due to their damped *q*_AC_ functions, cscLH23t-mBR, cscLH23t-mBR-P, and cscLH22t perform essentially identically as cLH20t (1.6%), on which they are based. This shows that for the statistically relevant, typically not very strongly correlated systems dominating NS372 (as confirmed by the applicability of double hybrids), the damped scLHs provide very little change in either direction, while the effects of the undamped scLHs are more pronounced. Statistically, the latter provide an overall slight improvement. An examination of *q*_AC_(**r**) will be provided further below, also for cases not contained in the statistical analyses. We note that the undamped scLHs perform even better than cLH20t for ^1^H shieldings.

The cωLH22t RSLH provides an overall performance more in line with cLH14t-calPBE or the specialized KT2 (rel. MAEs 2.0–2.1%), somewhat superior to RSHs such as ωB97X-V and cωB97M-V (2.3 and 2.5%, respectively). Importantly, all of these functionals still outperform many others evaluated in ref (26).

As for most of the functionals discussed there, the aggregate rel. MSE and that for most subsets remains negative, except in some cases for ^1^H. Interestingly, the undamped cscLH21t-SVWN-m has a particularly small negative rel. MSE. The aggregate rel. SDs for most of the functionals included here are below 2%. All of the scLHs exhibit values below 1.5%, where cscLH21t-SVWN-m (1.1%) almost approaches the top-performing DSD-PBEP86 (0.8%). A small rel. SD is important if we want to benefit from systematic error compensation in going from shieldings to relative shifts. The scLHs tend to benefit from this, albeit not as much as the TPSS-based mPSTS LH.^26^ The overall trends of the shifts do thus largely follow the shielding results, and we provide only a short discussion of shifts for NS372 in the Supporting Information (Section S3 and Figure S1, see also Table “NS372 Shifts” in the Supporting data).

The maximum percentage unsigned deviations (max. rel. AE) are particularly informative regarding the effects of the sc-corrections when comparing the scLHs with their underlying LH models (Figure 1): the scLHs (in particular the undamped ones) reduce the maximum deviations in particular for ^31^P/^33^S (in particular due to PN, see below), ^15^N (also PN), but to some extent even for ^13^C. The largest deviations for ^17^O are also reduced for the damped scLHs but actually *increased* for the undamped scLHs. The larger ^17^O outliers for the undamped models are due to OF_2_ (see below). Interestingly, compared to cLH20t, cscLH22ta improves upon the largest deviations for ^19^F but actually deteriorates matters somewhat for ^1^H. The damped scLHs do not show any individual outliers above ca. 9%, which is comparable to cB97M-V and significantly outperformed only by DSD-PBEP86 (6.5%). The cωLH22t RSLH shows somewhat larger outliers compared to LH20t for a number of nuclei, comparable to the RSHs. It appears that the inclusion of long-range EXX admixture causes too large paramagnetic contributions for some of the more sensitive cases.

### 5.2 Sensitive Main-Group Shielding Cases

The NS372 set is dominated by molecules exhibiting only weak correlations. Yet, we could see from the maximum percentage absolute errors above that there are also some cases with larger static correlation contributions. Additionally, we excluded a few systems (O_3_, F_3_^–^, and BH for ^1^H) from the statistical evaluations altogether. Such more sensitive cases are of particular significance for evaluating scLHs, and we scrutinize them more closely in the following. Indeed, we can identify such cases most straightforwardly from larger differences between the results of scLHs and their underlying uncorrected LHs. scLH21ct-SVWN-m^39^ is most closely related to LH12ct-SsirPW92,^62^ while the four other scLHs are all based on LH20t.^42^ Among the molecules chosen, O_3_, F_3_^–^, BH, PN, and SO_2_ had also received closer scrutiny in our previous study.^26^ The former two systems are clear multireference cases, and the others exhibit very large paramagnetic contributions. Previous studies^26,104^ suggest still notable changes from the CCSD(T) to CCSDT level for the most extreme cases of O_3_ and F_3_^–^ (50–77 ppm for O_3_ and 31–99 ppm for F_3_^–^). Instead of the usual CCSD(T)/pcSseg-3 reference data used for NS372 overall, we use CCSDT/pcSseg-3 data^26^ for F_3_^–^ and extrapolated CCSDT data (see Section S2 in the Supporting Information for details) for O_3_.

**Figure 2:**
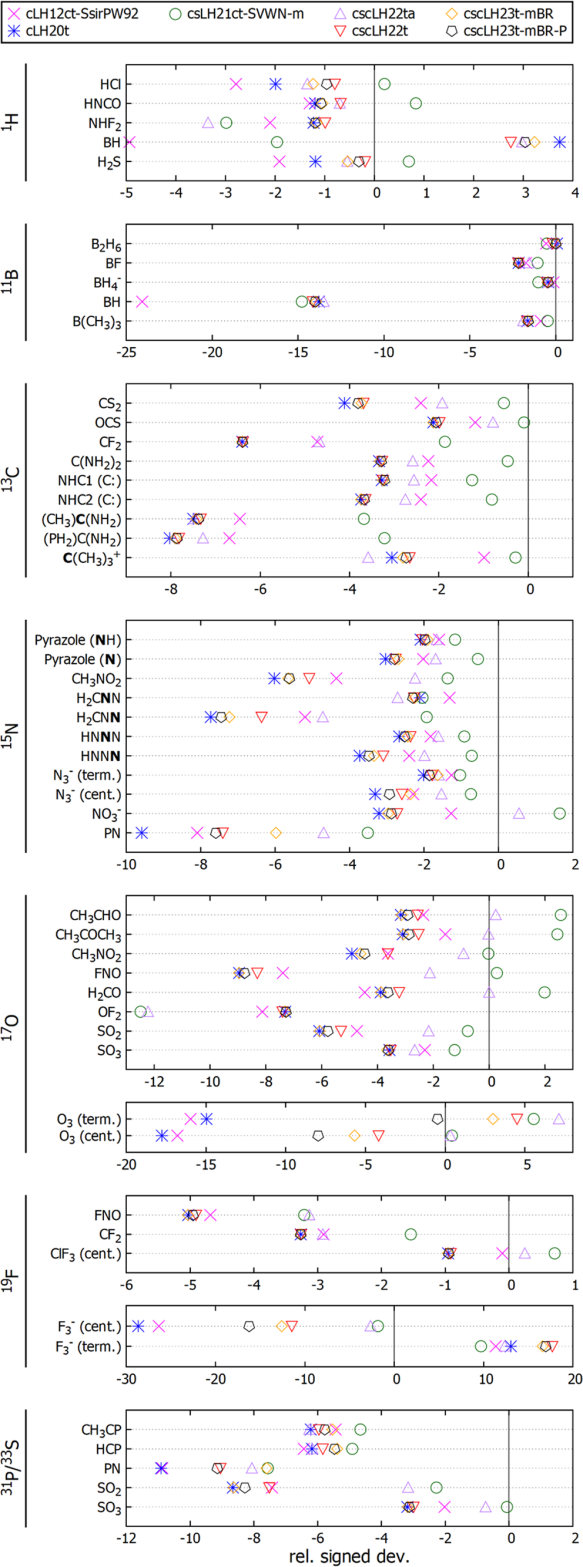
Signed percentage deviations relative to CCSD(T) or CCSDT (O_3_, F_3_^–^) shielding reference data with different functionals for molecules most sensitive to sc-corrections for different main-group nuclei.

Figure 2 compares the signed percentage deviations from the reference data for the five scLHs with those of cLH20t and cLH12ct-Ssir-PW92. Apart from the cases discussed already in previous work,^26^ the “sensitive cases” include not only the expected ^15^N, ^17^O, and ^31^P/^33^S shieldings for molecules with larger paramagnetic contributions but also, e.g., some ^13^C nuclei with low coordination numbers (carbenes, carbocations, CS_2_). As expected from the statistical evaluations above, the undamped scLH models have a far larger impact than the damped ones. The latter typically remain relatively close to the cLH20t results, with some exceptions: the damped cscLH22t provides already substantial corrections to the shieldings of both nuclei in PN (where cscLH23t-mBR provides particularly substantial corrections), as well as to all shieldings for the most clear-cut multireference systems O_3_ and F_3_^–^. In the remaining cases, the corrections from the damped scLHs remain comparably small, while the effects of the undamped models (cscLH22ta and cscLH21ct-SVWN-m) are much more substantial. In a few cases, these latter models overcorrect the results from the underlying LHs. This holds in particular for the simpler LDA-based cscLH21ct-SVWN-m functional. Examples can be seen with some ^1^H shieldings, with the ^15^N shielding of NO_3_^–^, and with the ^17^O shieldings of CH_3_CO, CH_3_COCH_3_, and H_2_CO.

Only in very few cases do the sc-corrections generally deteriorate the agreement with the reference data (e.g., for the ^17^O shielding in OF_2_ or the ^1^H shielding in NF_2_H). In most of the cases covered in Figure 2, however, the undamped models move the shieldings closer to the reference data, and then the simpler cscLH21ct-SVWN-m provides the best agreement, which is reflected also in the statistical improvements discussed above. These evaluations provide a picture where the damping factors in the damped scLH models are effective in retaining the underlying excellent performance of, e.g., cLH20t for weakly correlated situations (as found previously also for energy quantities^40,41^), but they fail to provide sufficiently large corrections in the intermediate range of moderately large static correlations. The undamped models provide much larger corrections but can overshoot in some cases, and they can deteriorate the overall performance for weakly correlated situations, as shown in the example of GMTKN55 energetics.^40,41^ That is, while scLHs provide a substantial step outside the usual “zero-sum game”,^35,36^ some trade-offs between the performances for weakly and strongly correlated situations remain.

### 5.3 Analyses of the Effects of the *q*_AC_(**r**) Strong-Correlation Factors for Selected Examples

**Figure 3:**
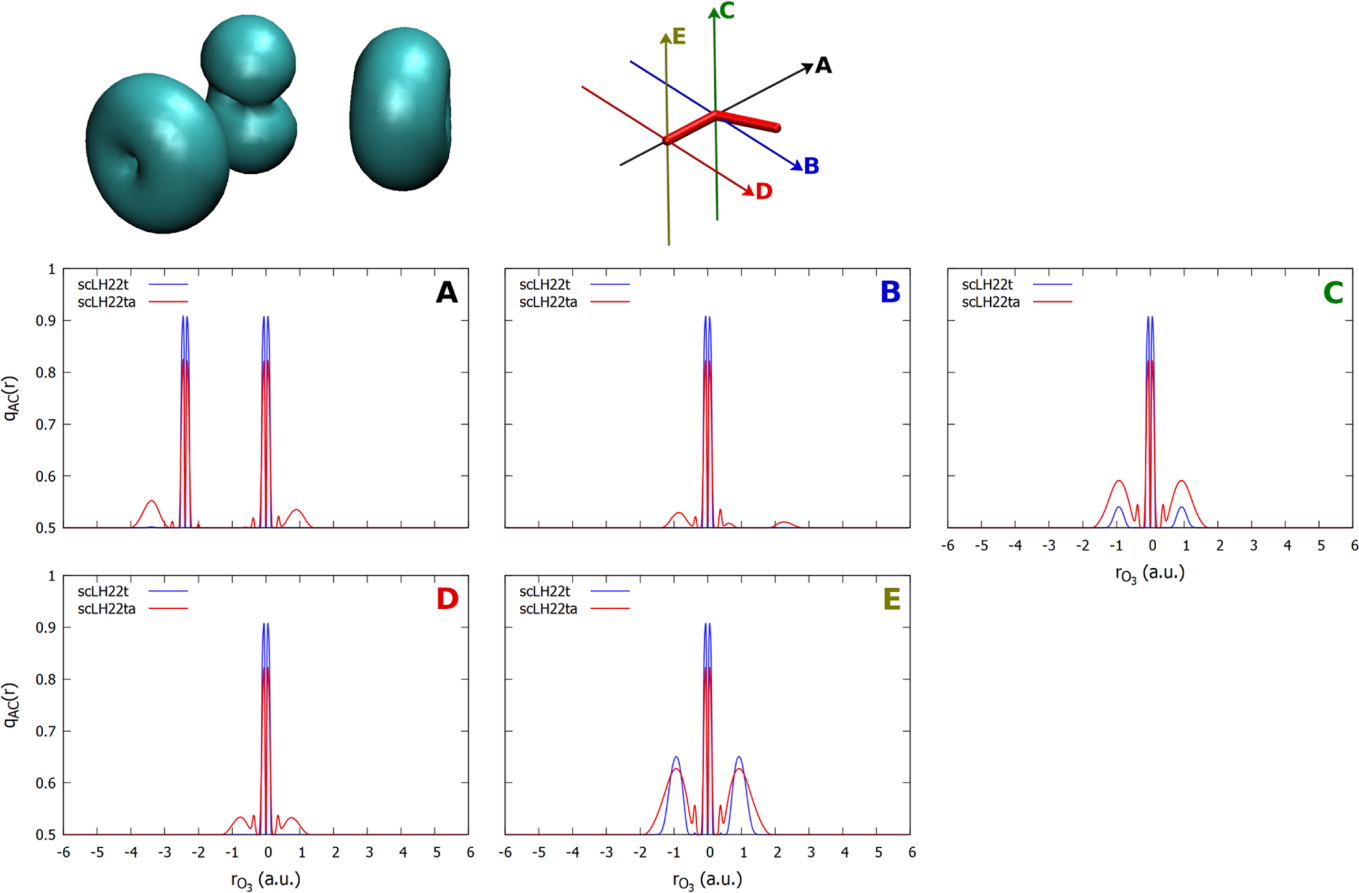
Top: plot of the FOD (isosurface = 0.002 au) and orientation of the *q*_AC_ plots for O_3_. Bottom: one-dimensional plots of *q*_AC_ with the scLH22t and scLH22ta functionals along the indicated directions.

**Figure 4:**
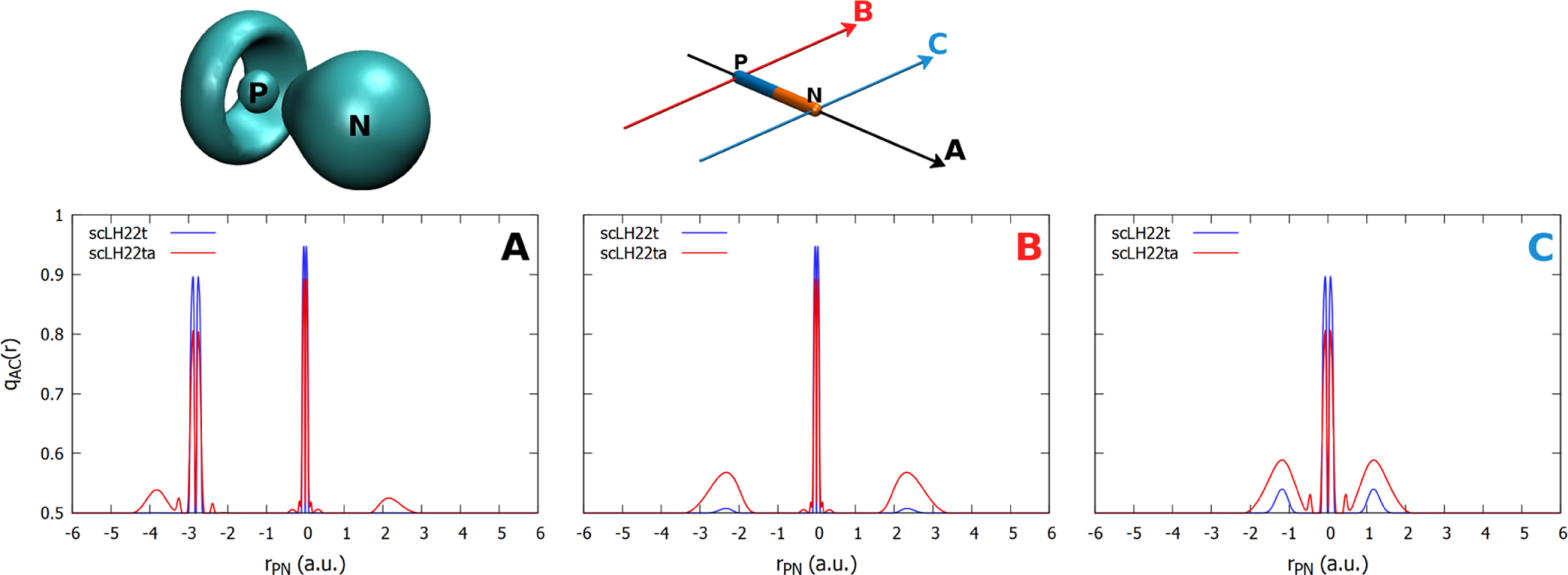
Top: plot of the FOD (isosurface = 0.002 au) and orientation of the *q*_AC_ plots for PN. Bottom: one-dimensional plots of *q*_AC_ with the scLH22t and scLH22ta functionals along the indicated directions.

To better understand the effects of the sc-factors on the shieldings of the more sensitive cases, it is instructive to plot the *q*_AC_(**r**) function. Figures 3 and 4 provide one-dimensional plots along different directions for O_3_ and PN, respectively. We should note that *q*_AC_(**r**) can also be incorporated into the LMF of the given LH to provide an sc-corrected LMF. For scLHs with damping factors based on LH20t, this is particularly informative, as the parameters of the underlying LH remain unchanged. We should note that, e.g., a deviation of *q*_AC_(**r**) from 0.5 by 0.1 at a point in space corresponds to a local reduction of the EXX admixture at this point by 0.2 (we note in passing that *q*_AC_(**r**) also enhances the dynamical correlation contributions by the same amount, cf. eq 16). We focus, in particular, on the comparison between the damped scLH22t and the undamped scLH22ta. We also provide FOD isosurface plots for comparison. Like *q*_AC_(**r**), FOD is a real-space function indicating regions of static correlation in a molecule, albeit obtained from fractional-occupation calculations (i.e., Fermi-smearing) at elevated temperatures.^102^ Our experience so far is that FOD and the different forms of *q*_AC_(**r**) indeed produce relevant magnitudes in similar regions of space. As we currently cannot plot *q*_AC_(**r**) three-dimensionally, the FOD isosurface plots provide additional orientation.

For O_3_, one of the most clear-cut strong-correlation cases (Figure 3), we see the most pronounced FOD shapes perpendicular to the O–O bonds, largely out-of-plane for the central oxygen atom and circular for the terminal ones. We disregard in the following the sharp features in *q*_AC_(**r**) close to the nuclei. These are artifacts of the construction of *q*_AC_(**r**) for these models and do not affect the results notably.^40^ In fact, these artifacts are removed in other models not based on exchange-hole normalization, e.g., for scLH23t-mBR.^41^ We are thus interested in regions further removed from the nuclei, as the paramagnetic shielding contributions depend mainly on the valence orbitals (see, e.g., Chapter 18 in ref (1). for a discussion). For the undamped scLH22ta, deviations of *q*_AC_(**r**) from the weak-correlation value of 0.5 are visible in all directions but are most pronounced perpendicular to the molecular plane for both central and terminal oxygen atoms. For the damped scLH22t, the features in *q*_AC_(**r**) are markedly smaller and remain significant only perpendicular to the molecular plane, in particular in the terminal position. In this extreme case, both functionals still provide substantial corrections to the oxygen shieldings in O_3_ (cf. Figure 2), but the undamped scLH22ta clearly produces larger effects (see above). We find smaller differences between the damped and undamped *q*_AC_(**r**) functions for F_3_^–^ (see Figure S2 in the Supporting Information). In this case, the main features for both scLHs occur along the bond axis around both central and terminal fluorine atoms, consistent with the shape of the FOD.

For PN, which is not as clearly a multireference case as O_3_ or F_3_^–^, the FOD isosurface plot shows significant features around both atoms, producing a ring shape around phosphorus and significant amplitude around the nitrogen atom (Figure 4). The plots of *q*_AC_(**r**) give the largest contributions around the nitrogen atom perpendicular to the bond axis. All features are significantly reduced for the damped scLH22t compared to scLH22ta, and only the perpendicular ones around nitrogen remain significant. scLH22t and the other damped models still provide significant corrections to both ^15^N and ^31^P shieldings (Figure 2), but less so than the undamped scLHs. For many other systems, the damped scLHs remain relatively close to the underlying cLH20t shielding results.

Among the few cases where this may be desirable, as the sc-corrections deteriorate agreement with the reference data (see above), we single out the ^17^O shielding in OF_2_ (Figure 2). Figure S3 in the Supporting Information shows that compared to scLH22ta, the deviations of *q*_AC_(**r**) from 0.5 are reduced by the damping factor of scLH22t also in this case. We cannot identify any notable aspects in these plots that would explain why here the sc-corrections do not improve the shieldings. Overall, the features in *q*_AC_(**r**) are notably smaller than, e.g., for O_3_ (see above).

### 5.4 Statistical Evaluations for the TM70 Benchmark

**Figure 5:**
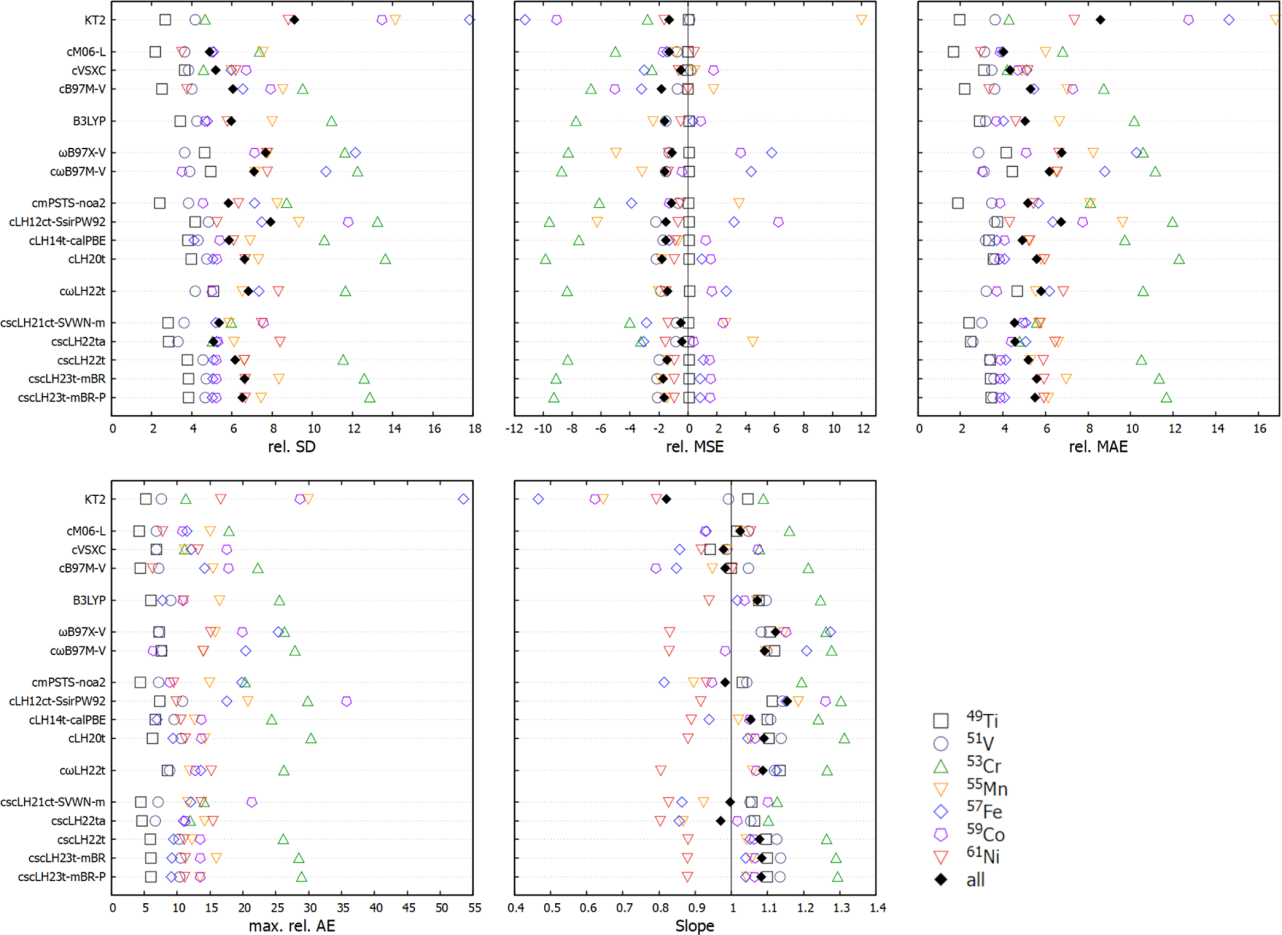
Statistical comparison of different functionals for the TM70 3d-nucleus NMR shift benchmark.^27^ Percentage relative deviations and the slope of the regression lines are shown. Results with scLHs and the ωLH22t RSLH are compared with data for selected other functionals from ref (27).

As discussed previously,^27^ the 3d-nucleus chemical shifts of the TM70 benchmark are generally a more pronounced challenge for DFAs than typical main-group shielding/shift sets. While the best-performing functionals can clearly provide percentage MAEs below 1.5% for NS372 (see above), for TM70, none of the functionals achieves values below 4%. The plot ranges of the statistical measures in Figure 5 are appropriately adapted compared to the main-group set. Note that here, MP2 or DH functionals have been found to be completely inadequate^27^ and are not included in the best-performing functionals from the previous study shown in Figure 5.

We start again with a bird’s eye view of the rel. MAEs. The best-performing functionals of the initial study gave values near or slightly below 5%: cM06-L (4.0%), cVSXC (4.3%), cLH14t-calPBE (4.9%), B3LYP (5.0%), cLH07t-SVWN (5.1%), cmPSTS (5.2%), and cB97M-V (5.3%). Here, a larger prefactor of a common t-LMF and the related enhanced EXX admixture deteriorates performance somewhat for LHs, and therefore, cLH20t (5.6%) falls slightly behind the best-performing models (simpler LHs with similarly large prefactors provide still larger errors, e.g., cLH12ct-SsirPW92 6.7%). For cLH20t, this stems predominantly from the larger errors caused by larger EXX admixtures for the ^53^Cr shifts of high-oxidation-state chromium complexes. The ^53^Cr subset has been known earlier to be better reproduced by “pure” GGA functionals than with hybrid functionals like B3LYP,^21^ and this had been confirmed for LHs.^27^ For the simpler cLH12ct-SsirPW92, larger deviations are also seen for the ^55^Mn and ^59^Co subsets.

Introducing the scLHs, we find that the two undamped cscLH22ta and cscLH21ct-SVWN-m (both 4.6%) are now the best-performing LHs overall, outperforming cLH14t-calPBE in spite of its lower t-LMF prefactor. Notably, these two scLHs are currently also the top-performing LHs for NS372 (see above), whereas among regular LHs the top position shifted between NS372 and TM70 performances.^26,27^ The damped scLH models improve less or not at all over cLH20t, with cscLH22t (5.3%) providing the overall best performance. The performance of the cωLH22t RSLH (5.9%) is slightly behind cLH20t but is better than, e.g., those of the cωB97M-V and ωB97X-V RSHs (6.2%, 6.8%).

A closer examination of the rel. MAEs for individual subsets shows that the improvements of the undamped scLHs over cLH20t are indeed dominated by the improvement of the ^53^Cr shifts, and the less pronounced improvement for this subset by the damped models is the reason that they also do not improve the aggregate performance that much. While cscLH22ta improves over the cLH20t results also somewhat for ^49^Ti and ^51^V, i.e., for the early 3d nuclei, it deteriorates agreement slightly for ^57^Fe and ^59^Co, thereby preventing the model from achieving an even better aggregate performance. The simpler LDA-based cscLH21ct-SVWN-m functional improves notably over related LHs like cLH12ct-SsirPW92 for all subsets except for ^61^Ni (Figure 5).

These trends are also reflected in the maximum percentage absolute errors (Figure 5), which for most hybrid functionals are dominated by ^53^Cr (cLH12ct-SsirPW92 has an even larger deviation arising from ^59^Co). The damped scLHs reduce these maximum deviations significantly. In particular, csLH22ta has no deviation of more than 15.5%, which is even better than the otherwise “best” functionals in this category, cM06-L 17.9% and cVSXC 17.6%. All other functionals exhibit cases with deviations above 20%, in most cases significantly so. Consequently, the undamped scLHs are also among the functionals with the lowest percentage SD (again, together with cM06-L and cVSXC).

The high sensitivity of the ^53^Cr subset has been analyzed in detail in ref (27) and could be traced back to its exclusive makeup from complexes with chromium in its + VI and 0 oxidation states (experimentally accessible Cr complexes in other oxidation states tend to be paramagnetic). It is, in particular, the shieldings of Cr(+VI) complexes that are deteriorated by EXX admixture in a hybrid functional and even partly by current contributions with meta-GGAs.^27^ That is, even pure GGA functionals overestimate the slope of the regression line, which is then further increased by EXX admixture. Semilocal functionals tend to overestimate the shift differences (around 2000 ppm) between the Cr(+VI) and Cr(0) subsets already by ca. 250–300 ppm, and current contributions in meta-GGAs or moderate EXX admixtures in hybrid functionals increase this gap further by up to 850 ppm, larger EXX admixtures by even more. For large EXX admixtures, the generalized Kohn–Sham wave functions even exhibit triplet instabilities, and we speculated that remnants of this behavior are still important for the shieldings obtained with lower admixtures.^27^ Notably, these instabilities are most pronounced in the higher oxidation state, where bonding of spatially contracted metal 3d orbitals with the ligand orbitals is hampered by Pauli repulsion with the 3*s*/3p semicore–shell, generating a “stretched-bond situation”.^105^ The same holds, to lesser extents, also for the vanadium and titanium complexes, where the shift range is also dominated by high-oxidation-state complexes with d^0^ configurations.

As discussed above, the *q*_AC_(**r**) factors of the scLHs decrease locally the EXX admixtures in the valence-shell region upon detection of strong-correlation effects, again more so for the undamped than for the damped models. This reduces the overestimated shift ranges for the ^53^Cr (as well as ^49^Ti and ^51^V) systems, also reducing the relative MAEs, SDs, slopes, and maximum deviations. In fact, with cscLH21ct-SVWN-m and cscLH22ta, all of those values for ^53^Cr tend to be closer to GGA results. The other subsets that tend to determine the overall performance of a given functional for TM70 are ^55^Mn, ^57^Fe, and ^59^Co. Here, moderate EXX admixtures in hybrid functionals or current terms for meta-GGAs tend to be beneficial rather than detrimental.^27^ More subtle aspects of static correlation and self-interaction errors appear to render analysis more complicated for these subsets. As discussed above, for the main-group cases, the damped *q*_AC_(**r**) factors provide much smaller corrections to the underlying cLH20t than the undamped ones. Improvements in relative deviations tend to be limited, and they are above 1% for only five ^55^Mn cases and above 2% only for Mn(NO)_3_CO and MnO_4_^–^.

### 5.5 Sensitive Transition-Metal Shift Cases

The overall larger error margins and the triplet instabilities for elevated EXX admixtures in some of the transition-metal subsets^27^ suggest significant effects of the sc-corrections. Interestingly, only very few cases of this nature are found for the ^49^Ti, ^51^V, and ^59^Co subsets, where also the statistical effects of the scLHs were smaller (see above). The deviations between undamped scLHs and the corresponding underlying LHs exceed 3% in just 6 out of 31 of these complexes, [Co(H_2_O)_6_]^3+^, [VF_5_] (with deviations exceeding 5%), [V(CO)_5_N_2_]^−^, [V(CO)_6_]^−^, [VOMe_3_], and [Co(acac)_3_]. In the ^61^Ni subset, 6 out of 9 complexes give deviations above 3%, but none above 5%. Even for this subset, the impact on the statistical data is low (see above). The damped scLHs generally provide changes below 1% for these subsets.

We, therefore, concentrate on some of the more sensitive examples from the overall more affected ^53^Cr, ^55^Mn, and ^57^Fe subsets and select, in each case, a few complexes on the shielded and the deshielded end of a given subset. In contrast to the NS372 set above, we only have relative NMR shifts as reference data here. This generates the problem that sc-effects for the reference standard may mask the effects on the shielding of the complex in question. On the other hand, a focus on absolute shieldings will provide us only with insight into the changes at hand, without clear-cut knowledge of whether a given change is beneficial or not. Table 2, therefore, provides both absolute shielding results and relative shifts obtained with the Y-intercept method (the three experimental reference standards are included).

**Table 2:** Absolute Shieldings and Relative Shifts with the Y-Intercept Method (in ppm) of Selected Complexes of the ^53^Cr, ^55^Mn, and ^57^Fe Subsets of TM70^a^

shieldings		c12sir^b^	cLH20t	cωLH22t	csc21^b^	cscLH22ta	cscLH22t	csc23mBR^b^	csc23mBR-P^b^
^53^Cr	[Cr(CO)_5_(PF_3_)]	–899.1	–990.9	–1013.1	–643.6	–703.9	–953.0	–989.8	–988.8
	[Cr(CO)_6_]	–861.5	–947.5	–969.7	–611.0	–666.9	–909.4	–946.4	–945.3
	[CrO_4_]^2–^	–3341.9	–3431.4	–3355.1	–2820.9	–2827.4	–3350.3	–3426.8	–3415.0
	[CrO_3_Cl]^−^	–3403.2	–3499.4	–3431.0	–2794.0	–2802.6	–3370.0	–3449.1	–3462.6
	[CrO_2_Cl_2_]	–3714.9	–3827.9	–3756.2	–3004.9	–3017.4	–3631.2	–3742.2	–3777.6
^55^Mn	[Mn(CO)_5_H]	–1544.6	–1688.8	–1674.6	–1109.9	–1192.5	–1684.2	–1708.5	–1718.6
	[Mn(CO)_5_]^−^	–1630.6	–1772.3	–1664.3	–1108.3	–1191.8	–1697.5	–1772.1	–1772.2
	[MnO_4_]^−^	–4832.8	–5003.5	–4864.2	–3730.9	–3791.0	–4834.5	–5100.3	–4968.5
	[MnCp(C_7_H_8_)]	–5956.4	–5514.9	–5516.5	–4442.0	–4304.6	–5461.2	–5482.7	–5482.5
^57^Fe	[Fe(CO)_3_(C_4_H_4_)]	–2004.0	–2162.5	–2043.9	–1242.3	–1363.1	–2138.7	–2175.6	–2175.2
	[Fe(CO)_5_]	–2421.5	–2607.6	–2472.4	–1732.0	–1854.7	–2589.7	–2606.7	–2608.2
	[Fe(CO)_3_(CH2CHCHO)]	–3868.5	–3999.9	–3913.3	–2775.7	–2930.4	–3981.4	–3999.5	–3999.1
	[FeCp_2_]	–4471.9	–4316.9	–4391.9	–3145.0	–3202.3	–4309.9	–4309.6	–4311.1

shifts		exp.	c12sir^b^	cLH20t	cωLH22t	csc21^b^	cscLH22ta	cscLH22t	csc23mBR^b^	csc23mBR-P^b^
^53^Cr	[Cr(CO)_5_(PF_3_)]	–1824	–2379.4	–2385.3	–2298.3	–2056.8	–2009.0	–2297.0	–2346.9	–2355.0
	[Cr(CO)_6_]	–1795	–2417.0	–2428.6	–2341.7	–2089.5	–2046.0	–2340.6	–2390.3	–2398.5
	[CrO_4_]^2–^	0	63.4	55.3	43.7	120.5	114.5	100.3	90.0	71.3
	[CrO_3_Cl]^−^	112	124.7	123.3	119.6	93.5	89.7	120.0	112.3	118.9
	[CrO_2_Cl_2_]	261	436.4	451.8	444.8	304.5	304.5	381.2	405.4	433.8
^55^Mn	[Mn(CO)_5_H]	–2528	–3034.7	–2766.5	–2730.9	–2328.3	–2233.2	–2707.2	–2775.8	–2727.1
	[Mn(CO)_5_]^−^	–2780	–2948.7	–2683.1	–2741.1	–2330.0	–2234.0	–2694.0	–2712.1	–2673.5
	[MnO_4_]^−^	0	253.5	548.1	458.8	292.7	365.2	443.1	616.0	522.8
	[MnCp(C_7_H_8_)]	1077	1377.2	1059.5	1111.1	1003.7	878.9	1069.8	998.5	1036.8
^57^Fe	[Fe(CO)_3_(C_4_H_4_)]	–583	–563.6	–573.5	–588.1	–514.6	–525.7	–578.1	–564.2	–564.9
	[Fe(CO)_5_]	0	–146.1	–128.5	–159.5	–24.9	–34.2	–127.1	–133.0	–131.9
	[Fe(CO)_3_(CH_2_CHCHO)]	1274	1300.9	1263.8	1281.3	1018.8	1041.5	1264.7	1259.7	1259.1
	[FeCp_2_]	1532	1904.2	1580.9	1759.9	1388.1	1313.5	1593.2	1569.9	1571.0

a Experimental reference standards are [CrO_4_]^2–^, [MnO_4_]^−^, and [Fe(CO)_5_], respectively.

b Abbreviations for the functionals are c12sir = cLH12ct-SsirPW92, csc21 = cscLH21ct-SVWN-m, csc23mBR = cscLH23t-mBR, csc23mBR-P = cscLH23t-mBR-P.

As anticipated from the overall statistics (see above) and from the discussion for the NS372 benchmark set, results for the damped scLH models remain relatively close to those for the underlying LH, both for shieldings and shifts. The undamped models clearly exhibit larger effects. cscLH21ct-SVWN-m features large changes compared to the related LDA-based first-generation cLH12ct-SsirPW92, while cscLH22ta exhibits the largest changes compared to the underlying cLH20t.

Starting with the ^53^Cr examples (Table 2), the shifts would suggest the largest sc-effects for the two Cr(0) complexes. We can infer this again by comparing cscLH22t and cscLH22ta results. However, this is due to the entire subset and thus the Y-intercept being dominated by Cr(+VI) complexes. The absolute shieldings show larger sc-effects (e.g., differences between cscLH22ta and cscLH22t shieldings) for the Cr(+VI) complexes. Consequently, in the shift representation, the Cr(0) complexes appear somewhat more deshielded. Overall, this reduces the gap between the Cr(+VI) and Cr(0) shieldings and, thereby, the slope of the regression line (see above), providing better agreement with experimental observation for the undamped scLHs. sc-Effects for the damped scLHs are clearly much smaller but go in the same direction.

For the ^55^Mn cases (Table 2), matters become less clear-cut, as we do not have the clear distinction between the high and low oxidation-state examples we have for ^53^Cr. The Y-intercept of the first-generation cLH12ct-SsirPW92 is smaller than that for cLH20t or cωLH22t, and it is not reduced with cscLH21ct-SVWN-m. cscLH22ta reduces the Y-intercept notably compared to cLH20t or the damped scLHs. Looking at the absolute shieldings, we find a large increase (>1100 pm) from cscH22t to cscLH22ta for [MnCp(C_7_H_8_)] and [MnO_4_]^−^, much smaller ones (ca. 500 ppm) for [Mn(CO)_5_]^−^ and [Mn(CO)_5_H]. As for ^53^Cr, this reduces the gap between the most deshielded and most shielded cases by about 500 ppm. The resulting slope of the linear regression with the undamped scLHs is then somewhat too small, while that with the damped models is closer to unity.

For the ^57^Fe subset (Table 2), the experimental reference standard is the Fe(0) complex [Fe(CO)_5_]. Here, the relatively small deviations from a zero Y-intercept are clearly reduced further by the undamped scLHs. However, deviations from the experiment for the other three complexes are overestimated. Comparisons of the absolute shieldings with cscLH22t vs cscLH22ta show large sc-effects for [Fe(CO)_3_(CH_2_CHCHO)] (ca. + 1050 ppm) and [FeCp_2_] (ca. + 900 ppm) at the deshielded end of the scale and smaller effects for [Fe(CO)_5_] (+735 ppm) and [Fe(CO)_3_(C_4_H_4_)] (+776 ppm) on the shielded side. This compresses the shift range and reduces the linear regression slope also for this subset, somewhat too little for the damped scLHs and somewhat too much for the undamped ones (see also Figure 5).

**Figure 6:**
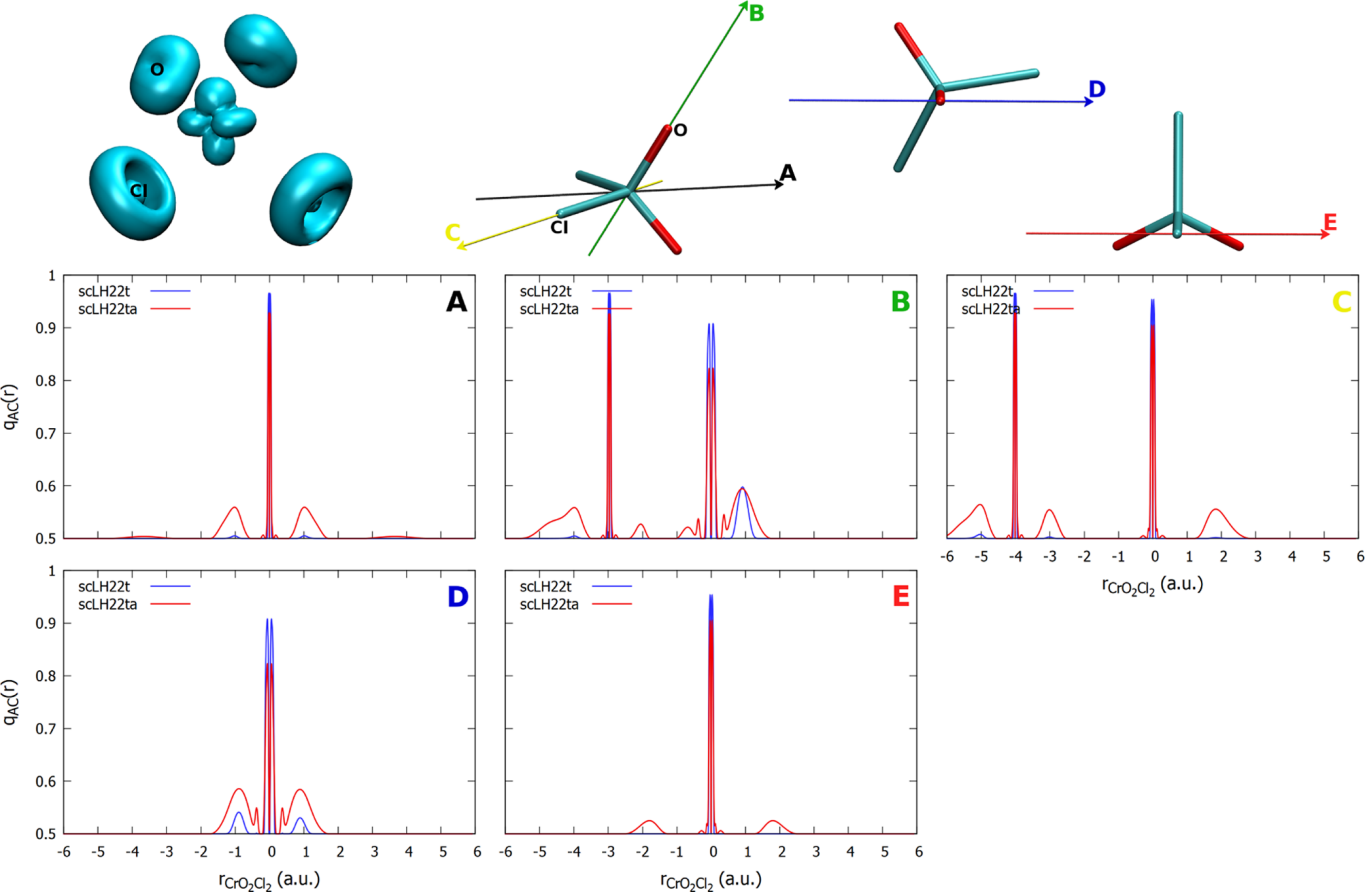
Top: plot of the FOD (isosurface = 0.002 au) and orientation of the *q*_AC_ plots for CrO_2_Cl_2_. Bottom: one-dimensional plots of *q*_AC_ with the scLH22t and scLH22ta functionals along the indicated directions.

**Figure 7:**
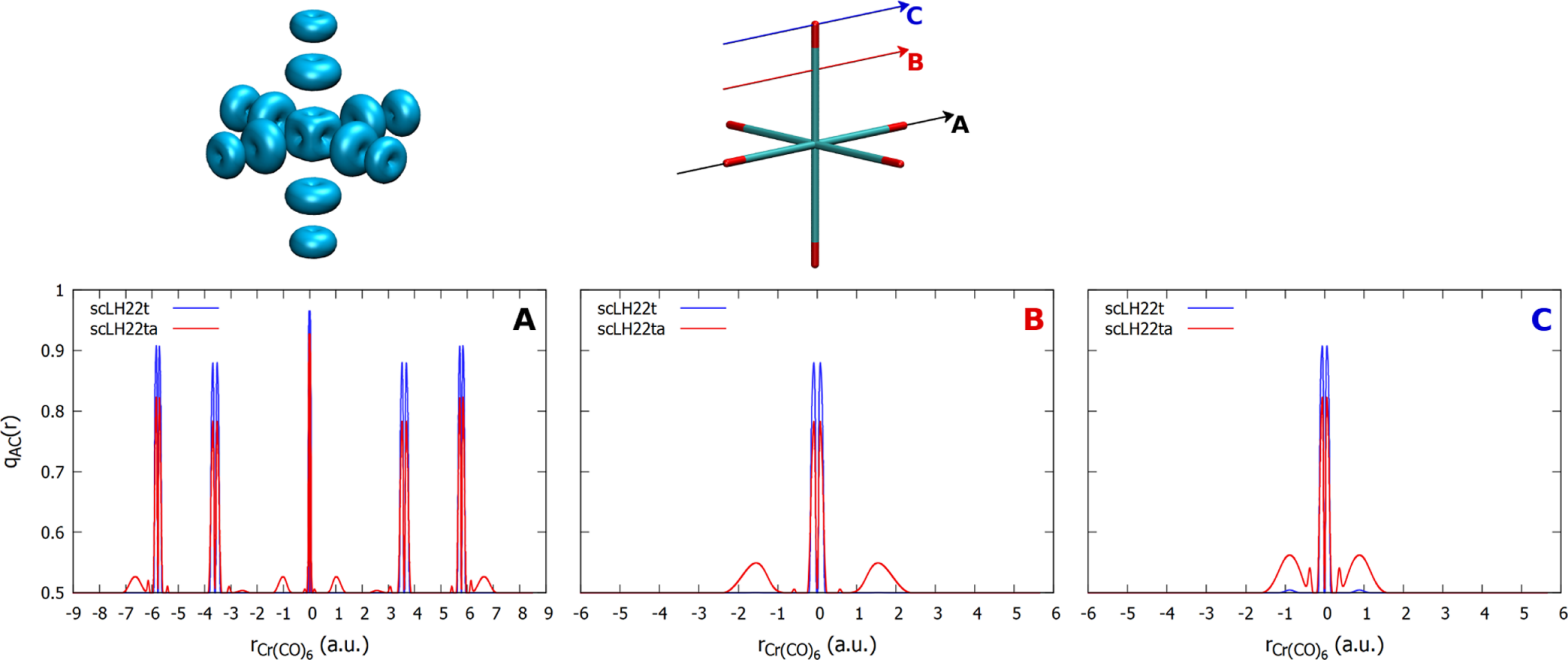
Top: plot of the FOD (isosurface = 0.002 au) and orientation of the *q*_AC_ plots for Cr(CO)_6_. Bottom: one-dimensional plots of *q*_AC_ with the scLH22t and scLH22ta functionals along the indicated directions.

The observation that within the ^53^Cr subset, the improvement by sc-corrections arises in particular from less deshielding of the Cr(+VI) complexes is analyzed further by comparing FOD and *q*_AC_ plots for the Cr(+VI) complex [CrO_2_Cl_2_] (Figure 6) and the Cr(0) complex [Cr(CO)_6_] (Figure 7). In both cases, we can observe the smaller features in *q*_AC_ for the damped scLH22t compared to the undamped scLH22ta. More importantly, the magnitude of the peaks is clearly larger for the Cr(+VI) complex. In this case, even the damped *q*_AC_ exhibits notable contributions at the oxo ligands (both along the bond and perpendicular to it, panels B and D in Figure 6). Features near the metal or chlorine atom are smaller and limited to the undamped *q*_AC_. For the Cr(0) complex, the damped *q*_AC_ has almost no contributions, and the undamped one has also much smaller ones, mostly at the carbon and oxygen atoms perpendicular to the bonds (Figure 7). The FOD plots also confirm more extended static correlation regions for [CrO_2_Cl_2_] than for [Cr(CO)_6_], consistent with sc-corrections becoming more important for the ^53^Cr shielding in the former complex and generally for the Cr(+VI) d^0^ than for the Cr(0) d^6^ species.

## 6 Conclusions

We have reported here the implementation and first evaluation for the computation of NMR shieldings/shifts of two new classes of state-of-the-art density functionals, both based on the exact-exchange-energy density. That is, our previous shielding implementation for local hybrid functionals has been extended to strong-correlation-corrected local hybrids as well as to range-separated local hybrids. The evaluation of two large and diverse test sets of both main-group and transition-metal shieldings and shifts has shown the appreciable potential of strong-correlation-corrected functionals to deal with difficult cases involving strong correlations. Within the less challenging NS372 main-group benchmark, only a relatively small subset of molecules benefits substantially from such corrections. We found that only so-called undamped variants of the strong-correlation terms (scLH22ta and scLH21ct-SWVN-m) are currently able to fully tap this potential and provide the overall best performance of a local hybrid for main-group shieldings. The damping factors introduced previously to retain the full accuracy of the underlying LH20t functional for weakly correlated situations prevent the resulting functionals (scLH22t, scLH23t-mBR, scLH23t-mBR-P) from providing the necessary corrections for systems with intermediate-strength static correlation effects. On the other hand, models without damping can overcorrect in some other cases while still providing overall statistically relevant improvement across a given subset. But then other data, e.g., the energetics of the GMTKN55 main-group test suite, may be somewhat deteriorated. These results show that the current variants of strong-correlation-corrected functionals have not yet fully escaped the trade-offs between weakly and strongly correlated cases but should be viewed as important steps on the way.

This conclusion is reinforced by the evaluations for the TM70 3d transition-metal shift benchmark, which exhibits a far larger number of systems with crucial static correlation effects. The undamped scLH22ta and scLH21ct-SVWN-m provide substantial improvements for sensitive shift cases, in particular for the ^53^Cr subset, where incipient triplet instabilities for high-oxidation-state Cr(+VI) complexes usually disfavor the use of hybrid functionals. Some improvements are found also for the ^51^V and ^49^Ti subsets, while small deterioration can also be seen for other subsets. Nevertheless, these two functionals are currently the statistically best-performing local hybrids for the TM70 set as well, among the best-performing functionals overall. Again, the other three damped models provide less of an improvement, with scLH22t still being promising.

We have provided further analyses by plotting the *q*_AC_(**r**) strong-correlation factors for selected molecules. It is clear that the local reduction of exact-exchange admixture in regions in a molecule identified to exhibit strong correlations can have a substantial effect on the electronic structure and, thus, on the nuclear shielding of various nuclei. The damping factors in some models seem to reduce these areas too much in some cases, thereby limiting the effectiveness of the resulting functionals to improve agreement with the benchmark data in certain intermediate strong-correlation cases. Yet the damping factors are important to avoid double counting of nondynamical correlations in more weakly correlated situations. Further improvements in such functionals are expected to provide an even finer balance between the different types of systems.

The first evaluations of the ωLH22t range-separated local hybrid for nuclear shieldings did not improve performance over the related LH20t functional for nuclear shieldings. Yet the results still make this a promising functional to be used for NMR shift calculations. Such range-separated local hybrids have other advantages, as they exhibit the correct long-range asymptotic potential. Very recent extensions to strong-correlation-corrected range-separated local hybrids^48^ offer the potential for further extending the application areas and improving accuracy, including for NMR shifts and other spectroscopic quantities.

## Supporting Information Available

The Supporting Information provides explicit expressions for the perturbed potentials of LHs, RSLHs, and scLHs; a description of the extrapolated CCSDT reference data for O_3_ with the pcSseg basis-set family; a brief discussion of the performance of the evaluated functionals for relative shifts of the NS372 benchmark set; and additional plots of FODs and *q*_AC_ functions for OF_2_ and F_3_^–^ (PDF)
Full shielding and shift data and statistics with RSLH and scLH functionals and cLH20t for the NS372 and TM70 benchmark sets (XLSX)
