# Supplementary material for: Implementation and First Evaluation of Strong-Correlation-Corrected Local Hybrid Functionals for the Calculation of NMR Shieldings and Shifts
Source: J Phys Chem A. 2024 Mar 8;128(11):2253–71. doi: 10.1021/acs.jpca.3c08507 (PMC10961831; doi:10.1021/acs.jpca.3c08507)
Supplement: Supplementary file 1 — jp3c08507_si_001.pdf [file jp3c08507_si_001.pdf]

Supporting Information: Implementation and First Evaluation of Strong-Correlation-Corrected Local Hybrid Functionals for the Calculation of NMR Shieldings and Shifts

Caspar Jonas Schattenberg[†] and Martin Kaupp[*,‡]

†*Research Unit of Structural Chemistry & Computational Biophysics, Leibniz-Forschungsinstitut für Molekulare Pharmakologie (FMP), Robert-Roessle-Str. 10, 13125, Berlin, Germany*

‡*Technische Universität Berlin, Institut für Chemie, Theoretische Chemie/Quantenchemie, Sekr. C7, Straße des 17. Juni 135, D-10623, Berlin, Germany*

E-mail: martin.kaupp@tu-berlin.de

Contents

S1 The perturbed potentials S3

S2 Extrapolated GIAO-CCSDT reference data for the ^{17}O shieldings in the O_3 molecule S5

S3 NS372 relative shifts S6

S4 Additional plots of q_{AC} S8

References S9

S1 The perturbed potentials

The LH functional as given in Equation (1) results in the perturbed LH potential as derived in ref. S1:

$$\begin{aligned}
\left(V_{\mu\nu,\varsigma}^{\text{LH}}\right)^{\mathbf{B}} = &\sum_{\sigma}\int\left(\frac{\partial e_{\text{X},\sigma}^{\text{ex}}}{\partial D_{\mu\nu}^{\varsigma}}\right)^{\mathbf{B}}\text{d}\mathbf{r} \\
&-\sum_{\sigma}\int(1-g)\cdot\left(\frac{\partial e_{\text{X},\sigma}^{\text{ex}}}{\partial D_{\mu\nu}^{\varsigma}}\right)^{\mathbf{B}}\text{d}\mathbf{r} \\
&+\sum_{\sigma}\int\left(\hat{d}_{\mu\nu,\varsigma}g\right)^{\mathbf{B}}\cdot e_{\text{X},\sigma}^{\text{ex}}\text{d}\mathbf{r} \\
&-\sum_{\sigma}\int\left(\hat{d}_{\mu\nu,\varsigma}g\right)^{\mathbf{B}}\cdot e_{\text{X},\sigma}^{\text{sl},*}\text{d}\mathbf{r} \\
&+\sum_{\sigma}\int(1-g)\cdot\left(\hat{d}_{\mu\nu,\varsigma}e_{\text{X},\sigma}^{\text{sl},*}\right)^{\mathbf{B}}\text{d}\mathbf{r} \\
&+\int\left(\hat{d}_{\mu\nu,\varsigma}e_{\text{C}}\right)^{\mathbf{B}}\text{d}\mathbf{r}
\end{aligned} \tag{S1}$$

The potential of the RSLH using the NDC/DC contribution as shown in Equation (13) in main text is given by

$$\begin{aligned}
\left(V_{\mu\nu,\varsigma}^{\text{RSLH}}\right)^{\mathbf{B}} = &\sum_{\sigma}\int\left(\frac{\partial e_{\text{X},\sigma}^{\text{ex},\omega}}{\partial D_{\mu\nu}^{\varsigma}}\right)^{\mathbf{B}}\text{d}\mathbf{r} \\
&-\sum_{\sigma}\int(1-g)\cdot\left(\frac{\partial e_{\text{X},\sigma}^{\text{ex,SR},\omega}}{\partial D_{\mu\nu}^{\varsigma}}\right)^{\mathbf{B}}\text{d}\mathbf{r} \\
&+\sum_{\sigma}\int\left(\hat{d}_{\mu\nu,\varsigma}g\right)^{\mathbf{B}}\cdot e_{\text{X},\sigma}^{\text{ex,SR},\omega}\text{d}\mathbf{r} \\
&-\sum_{\sigma}\int\left(\hat{d}_{\mu\nu,\varsigma}g\right)^{\mathbf{B}}\cdot\left(e_{\text{X},\sigma}^{\text{sl,SR},\omega}+G_{\sigma}\right)\text{d}\mathbf{r} \\
&+\sum_{\sigma}\int(1-g)\cdot\left(\hat{d}_{\mu\nu,\varsigma}\left(e_{\text{X},\sigma}^{\text{sl,SR},\omega}+G_{\sigma}\right)\right)^{\mathbf{B}}\text{d}\mathbf{r} \\
&+\int\left(\hat{d}_{\mu\nu,\varsigma}e_{\text{C}}\right)^{\mathbf{B}}\text{d}\mathbf{r}
\end{aligned} \tag{S2}$$

Finally, an scLH with the NDC/DC contribution of Equation (16) in main text provides a more involved perturbed potential, due to the presence of $q_{AC}(\mathbf{r})$:

$$
\begin{aligned}
\left(V^{\mathrm{sc}}_{\mu\nu,\varsigma}\right)^{\mathbf{B}} = & \sum_{\sigma}\int\left(\frac{\partial e^{\mathrm{ex},\omega}_{\mathrm{X},\sigma}}{\partial D^{\varsigma}_{\mu\nu}}\right)^{\mathbf{B}}\mathrm{d}\mathbf{r} \\
& 2\sum_{\sigma}\int(1-g)\cdot\frac{\partial q_{\mathrm{AC}}}{\partial e^{\mathrm{ex}}_{\mathrm{X},\varsigma}}\left(\frac{\partial e^{\mathrm{ex}}_{\mathrm{X},\varsigma}}{\partial D^{\varsigma}_{\mu\nu}}\right)^{\mathbf{B}}\cdot e^{\mathrm{sl},*}_{\mathrm{X},\sigma}\mathrm{d}\mathbf{r} \\
& -2\sum_{\sigma}\int(1-g)\cdot\frac{\partial q_{\mathrm{AC}}}{\partial e^{\mathrm{ex}}_{\mathrm{X},\varsigma}}\left(\frac{\partial e^{\mathrm{ex}}_{\mathrm{X},\varsigma}}{\partial D^{\varsigma}_{\mu\nu}}\right)^{\mathbf{B}}\cdot e^{\mathrm{ex}}_{\mathrm{X},\sigma}\mathrm{d}\mathbf{r} \\
& +2\int\frac{\partial q_{\mathrm{AC}}}{\partial e^{\mathrm{ex}}_{\mathrm{X},\varsigma}}\left(\frac{\partial e^{\mathrm{ex}}_{\mathrm{X},\varsigma}}{\partial D^{\varsigma}_{\mu\nu}}\right)^{\mathbf{B}}\cdot e_{\mathrm{C}}\mathrm{d}\mathbf{r} \\
& -2\sum_{\sigma}\int(1-g)\cdot q_{\mathrm{AC}}\cdot\left(\frac{\partial e^{\mathrm{ex}}_{\mathrm{X},\sigma}}{\partial D^{\varsigma}_{\mu\nu}}\right)^{\mathbf{B}}\mathrm{d}\mathbf{r} \\
& -2\sum_{\sigma}\int\left(\hat{d}_{\mu\nu,\varsigma}g\right)^{\mathbf{B}}\cdot q_{\mathrm{AC}}\cdot e^{\mathrm{sl},*}_{\mathrm{X},\sigma}\mathrm{d}\mathbf{r} \\
& +2\sum_{\sigma}\int\left(\hat{d}_{\mu\nu,\varsigma}g\right)^{\mathbf{B}}\cdot q_{\mathrm{AC}}\cdot e^{\mathrm{ex}}_{\mathrm{X},\sigma}\mathrm{d}\mathbf{r} \\
& +2\sum_{\sigma}\int(1-g)\cdot\left(\hat{d}_{\mu\nu,\varsigma}q^{\mathrm{sl}}_{\mathrm{AC}}\right)^{\mathbf{B}}\cdot e^{\mathrm{sl},*}_{\mathrm{X},\sigma}\mathrm{d}\mathbf{r} \\
& -2\sum_{\sigma}\int(1-g)\cdot\left(\hat{d}_{\mu\nu,\varsigma}q^{\mathrm{sl}}_{\mathrm{AC}}\right)^{\mathbf{B}}\cdot e^{\mathrm{ex}}_{\mathrm{X},\sigma}\mathrm{d}\mathbf{r} \\
& +2\int\left(\hat{d}_{\mu\nu,\varsigma}q^{\mathrm{sl}}_{\mathrm{AC}}\right)^{\mathbf{B}}\cdot e_{\mathrm{C}}\mathrm{d}\mathbf{r} \\
& +2\sum_{\sigma}\int(1-g)\cdot q_{\mathrm{AC}}\cdot\left(\hat{d}_{\mu\nu,\varsigma}e^{\mathrm{sl},*}_{\mathrm{X},\sigma}\right)^{\mathbf{B}}\mathrm{d}\mathbf{r} \\
& +2\int q_{\mathrm{AC}}\cdot\left(\hat{d}_{\mu\nu,\varsigma}e_{\mathrm{C}}\right)^{\mathbf{B}}\mathrm{d}\mathbf{r}
\end{aligned}
\tag{S3}
$$

S2 Extrapolated GIAO-CCSDT reference data for the ^{17}O shieldings in the O_3 molecule

We approximate the CCSDT reference value in a larger basis set from the pcSseg-(x) family, $\sigma_{\text{CCSDT,appr.}}^{\text{pcSseg-(x+1)}}$, by using the CCSDT value of the next lower rank of the basis-set hierarchy, corrected by the difference of CCSD(T) values with the larger and smaller basis as

$$\sigma_{\text{CCSDT,appr.}}^{\text{pcSseg-(x+1)}} = \sigma_{\text{CCSDT}}^{\text{pcSseg-(x)}} + (\sigma_{\text{CCSD(T)}}^{\text{pcSseg-(x+1)}} - \sigma_{\text{CCSD(T)}}^{\text{pcSseg-(x)}}). \tag{S4}$$

The extrapolated GIAO-CCSDT/pcSseg-3 reference values deviate by only 6.8 ppm ($O_{\text{term.}}$) and 26.8 ppm ($O_{\text{cent.}}$), respectively, from previously computed GIAO-CCSDT/qz2p shielding results.[S2] We decided to use the extrapolated values instead of the literature values for consistency with the chosen basis sets for NS372 and for F_3^- (see main text).

Table S1: Extrapolation of the CCSDT reference value for O_3 using pcSseg-X (X=1,2,3) basis sets.

		pcSseg-1		pcSseg-2		pcSseg-3	
		CCSD(T)	CCSDT	CCSD(T)	CCSDT	CCSD(T)	CCSDT
calc.	$O_{\text{term.}}$	-1177.5	-1256.9	-1178.7	-1246.5	-1200.0	–
	$O_{\text{cent.}}$	-704.9	-759.9	-736.0	-776.9	-760.7	–
extrapol.	$O_{\text{term.}}$				-1258.1		-1267.9
	$O_{\text{cent.}}$				-791.0		-801.5

S3 NS372 relative shifts

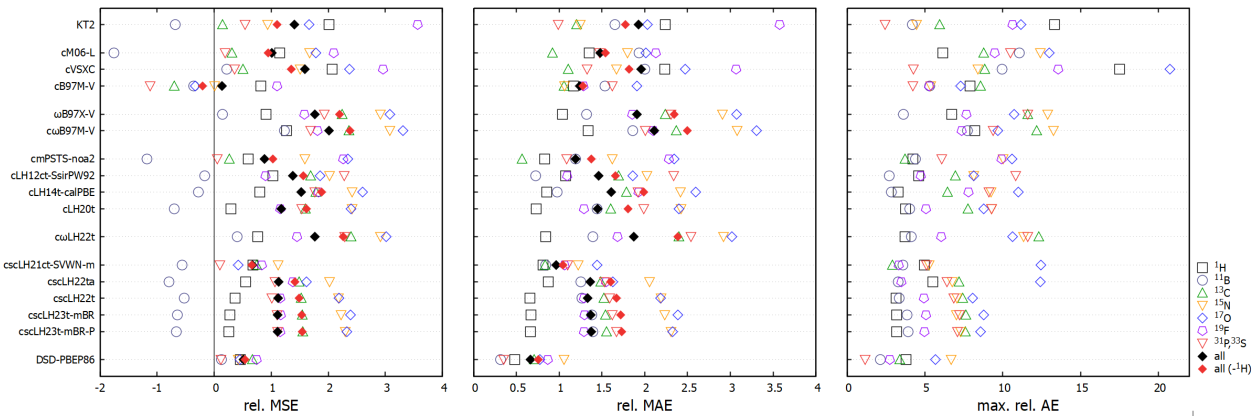

Figure S1: Statistical measures for relative NMR shifts of selected DFAs relative to the GIAO-CCSD(T)/pcSseg-3 reference data for the NS372 benchmark. Percentage deviations are shown.

While the discussion in the main text for NS372 has focused on absolute shielding constants and their deviations from the reference values, here we also provide a short statistical overview on relative shifts (see Equation (54) in main text). The latter involve some compensation between computational errors made for the compound in question and that made for the reference compound used to extract shifts for a given nucleus. When evaluating DFAs, some of them provide more effective error compensation than others, and a comparison can be instructive when selecting a DFA for applications. The computational reference standards employed for NS372 are: CH_4 (^{1}H, ^{13}C), BH_3 (^{11}B), NH_3 (^{15}N), H_2O (^{17}O), CF_4 (^{19}F), PH_3 (^{31}P) and H_2S (^{33}S). The error compensation involved can be seen from a narrower distribution of deviations compared to the shieldings discussed in main text (see Figure S1, which uses the same symbols and labels). We do not plot relative standard deviations, as these are almost identical to those for the absolute shieldings.

Starting with the aggregated percentage MAEs, we see that the best-performing functionals are still the same as for the absolute shieldings, but now cscLH21ct-SVWN-m (1.0 %) joins the best-performing double hybrid DSD-PBEP86 (0.7 %) as another functional that achieves the 1 % mark. This is followed by cmPSTS and cB97M-V (1.2 %). cmPSTS has been discussed before as a DFA example with a particularly effective error compensa-

tion for the relative shifts.[S3] The LH20t-based scLHs (cscLH22t, cscLH22ta, cscLH23-mBR, cscLH23-mBR-P) also all give low aggregate percentage MAEs of 1.3 – 1.4 %, not improving much over cLH20t (1.4 %). Here the damped cscLH22t switches ranks with the undamped cscLH22ta due to a very systematic behavior for ^{1}H shieldings (as also reflected by the smaller SD, see main text). When excluding ^{1}H shifts, the undamped model performs slightly better.

Due to the different sign convention of shifts compared to shieldings, the percentage mean signed deviations (middle panel) are mostly positive (except for the ^{11}B subset). The cB97M-V mGGA is an exception with an extremely small aggregate value (+0.1 %) due to negative values for several individual subsets (^{11}B, ^{13}C, ^{17}O, ^{31}P/^{33}S). The otherwise smallest positive aggregate deviations pertain to DSD-PBEP86 (0.5 %, only positive deviations in all subsets) and cscLH21ct-SVWN-m (0.7 %). The other scLHs remain overall close to the underlying cLH20t, with deviations comparable to cmPSTS.

The maximum unsigned deviations in each shift subset are also provided in Figure S1 (right panel). Except for a larger value for the ^{17}O subset arising from OF_2, the LDA-based cscLH21ct-SVWN-m would rival the best-performing DSD-PBEP86. The other undamped scLH, cscLH22ta, also exhibits a larger deviation above 12 % for ^{17}O but otherwise low maximum deviations. The damped scLHs have no value above 10 % in any of the subsets, nor does the underlying cLH20t. Other DFAs shown in the figure clearly exhibit a number of larger deviations.

S4 Additional plots of q_{AC}

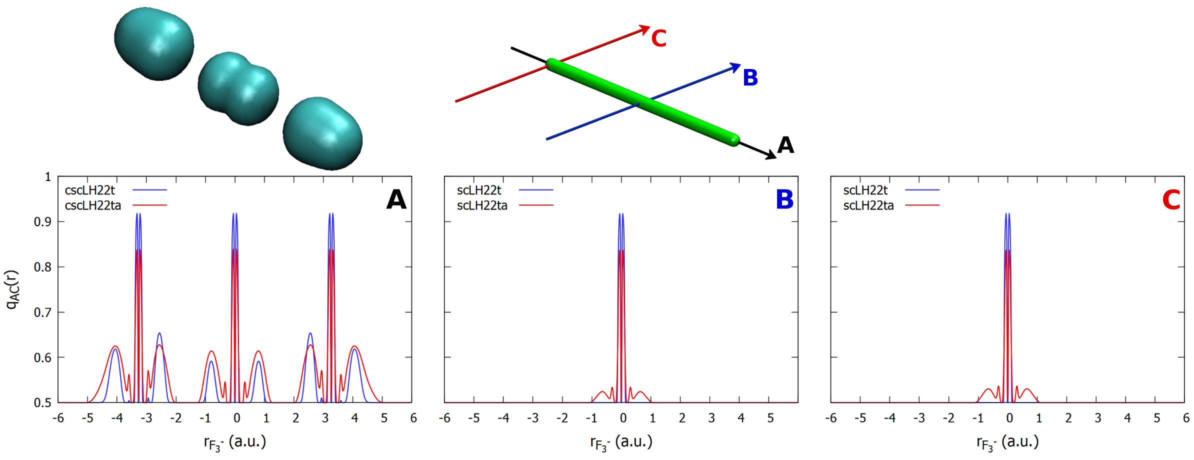

Figure S2: Top: plot of the FOD (isosurface=0.002 a.u.) and orientation of the q_{AC} plots for $F_3{}^-$. Bottom: one-dimensional plots of q_{AC} with the scLH22t and scLH22ta functionals along the indicated directions.

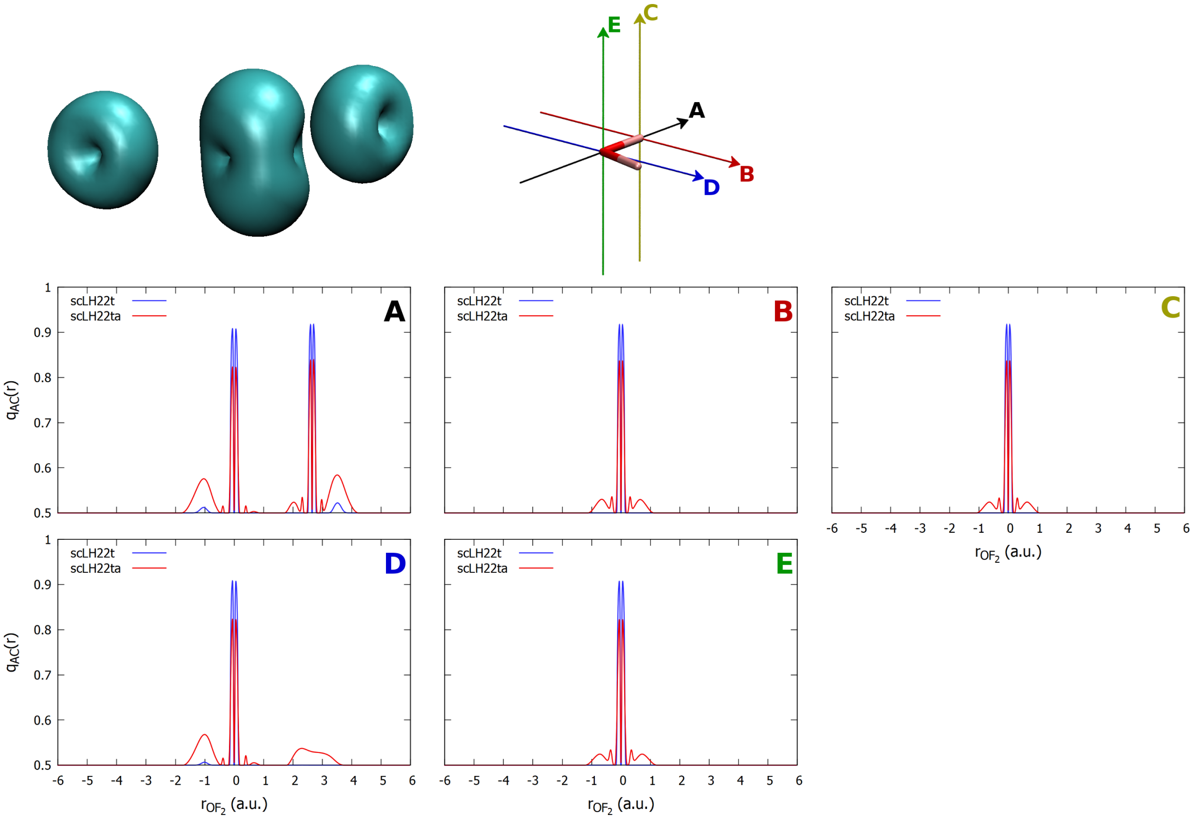

Figure S3: Top: plot of the FOD (isosurface=0.002 a.u.) and orientation of the q_{AC} plots for OF_2. Bottom: one-dimensional plots of q_{AC} with the scLH22t and scLH22ta functionals along the indicated directions.

References

(S1) Schattenberg, C. J.; Reiter, K.; Weigend, F.; Kaupp, M. An Efficient Coupled-Perturbed Kohn–Sham Implementation of NMR Chemical Shift Computations with Local Hybrid Functionals and Gauge-Including Atomic Orbitals. *J. Chem. Theory Comput.* **2020**, *16*, 931–943.

(S2) Gauss, J. Analytic second derivatives for the full coupled-cluster singles, doubles, and triples model: Nuclear magnetic shielding constants for BH, HF, CO, N_2, N_2O, and O_3. *J. Chem. Phys.* **2002**, *116*, 4773–4776.

(S3) Schattenberg, C. J.; Kaupp, M. Extended Benchmark Set of Main-Group Nuclear Shielding Constants and NMR Chemical Shifts and Its Use to Evaluate Modern DFT Methods. *J. Chem. Theory Comput.* **2021**, *17*, 7602–7621.
